# Decellularized extracellular matrix biomaterials for regenerative therapies: Advances, challenges and clinical prospects

**DOI:** 10.1016/j.bioactmat.2023.09.017

**Published:** 2023-10-04

**Authors:** Aleksandra A. Golebiowska, Jonathon T. Intravaia, Vinayak M. Sathe, Sangamesh G. Kumbar, Syam P. Nukavarapu

**Affiliations:** a Department of Biomedical Engineering, University of Connecticut, Storrs, CT, 06269, USA; b Department of Materials Science & Engineering, University of Connecticut, Storrs, CT, 06269, USA; c Department of Orthopaedic Surgery, University of Connecticut Health, Farmington, CT, 06032, USA

**Keywords:** Non-immune biomaterials and grafts, Native micro-environment, Bio-chemical cues, Chemotactic ability, Tissue-inks, Clinical therapies

## Abstract

Tissue engineering and regenerative medicine have shown potential in the repair and regeneration of tissues and organs via the use of engineered biomaterials and scaffolds. However, current constructs face limitations in replicating the intricate native microenvironment and achieving optimal regenerative capacity and functional recovery. To address these challenges, the utilization of decellularized tissues and cell-derived extracellular matrix (ECM) has emerged as a promising approach. These biocompatible and bioactive biomaterials can be engineered into porous scaffolds and grafts that mimic the structural and compositional aspects of the native tissue or organ microenvironment, both *in vitro* and *in vivo*. Bioactive dECM materials provide a unique tissue-specific microenvironment that can regulate and guide cellular processes, thereby enhancing regenerative therapies. In this review, we explore the emerging frontiers of decellularized tissue-derived and cell-derived biomaterials and bio-inks in the field of tissue engineering and regenerative medicine. We discuss the need for further improvements in decellularization methods and techniques to retain structural, biological, and physicochemical characteristics of the dECM products in a way to mimic native tissues and organs. This article underscores the potential of dECM biomaterials to stimulate *in situ* tissue repair through chemotactic effects for the development of growth factor and cell-free tissue engineering strategies. The article also identifies the challenges and opportunities in developing sterilization and preservation methods applicable for decellularized biomaterials and grafts and their translation into clinical products.

## Highlights

• Decellularized tissue-/cell-derived non-immune biomaterials with tissue-specific microenvironment, structure and mechanics
• Decellularized grafts potential to stimulate *in situ* tissue regeneration through chemotactic effects and presence of native GFs.
• The use of dECM biomaterials as “Tissue-Inks” for the bio-fabrication of engineered grafts.
• Sterilization/preservation importance and need to establish Good Manufacturing Practice standards for clinical translation
• Highlights recent developments, contemporary challenges, and decellularized-based grafts for regenerative therapies.

## Graphical abstract

**Figure:**
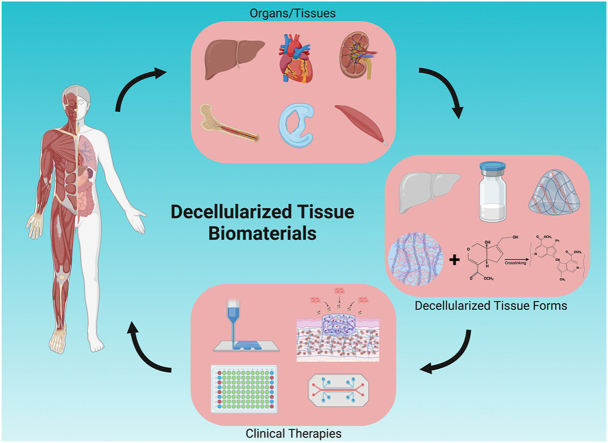

## 1 Introduction

Repair and regeneration of damaged, diseased or loss of tissues and organs remains a significant clinical challenge and costs more than $400 billion in US on a yearly basis [1]. Tissue engineering (TE) has emerged as an alternative to current treatment options for the development of engineered structures to improve and restore affected tissues and organs [2,3]. TE strategies involve the use of biomaterials along with relevant tissue-specific cells and/or growth factors (GFs). This triad work alone or in combination to provide the structural and biochemical cues to guide/regulate cell behavior and tissue development. Various natural and synthetic biomaterials have been utilized for the development of biodegradable three-dimensional (3D) and porous scaffolds that are capable of supporting cell in-growth and *de novo* tissue formation [[4], [5], [6]].

Tissue engineering efforts are focused on the development of biomimetic scaffold systems that can more closely replicate the complex microenvironment of native tissues and organs. Conventional TE scaffold fabrication methods including porogen-leaching, freeze-drying, phase-separation and electrospinning can generate 3D-porous structures with tissue-like microenvironment [7,8]. Advanced scaffold fabrication methods such as additive manufacturing helped to form structures with tunable porosity and gradient along the scaffold length to support tissue-tissue interfacial engineering [7,[9], [10], [11], [12]]. Significant progress has been made in the development of scaffolds that incorporate ECM-like structures with desired nano/micro topography that mimic the target tissue in terms of bio-chemical composition and mechanical performance [13,14]. Also, recent efforts resulted in more sophisticated scaffolds that can be built using synthetic ECM formed with bioactive domains [[15], [16], [17]]. However, despite this progress the engineered structures still lack the complex microenvironment and composition of the native ECM that is required to achieve optimal regenerative capacity [18].

The complex microenvironment of native tissues presents many challenges with recapitulating *in vivo* cell interactions and conditions to restore normal function, often varying with type of tissue, and state of health condition. The extracellular matrix (ECM) contains functional characteristics and structures including the complex establishment of proteins and matrix components of the target tissue/organ microenvironment such as collagen, proteoglycans, fibronectin, laminin, elastin, and other glycoproteins [19,20]. Moreover, the ECM also serves as a reservoir of essential GFs and signaling molecules and plays a vital role in regulating and maintaining tissue homeostasis, growth, differentiation, vascularization and maturation [21]. These properties of native ECM present challenges in reproducing the complex 3D ultrastructure with appropriate compositional arrangement and are often difficult to achieve with conventional fabrication methods and biomaterials [22].

**Fig. 1:**
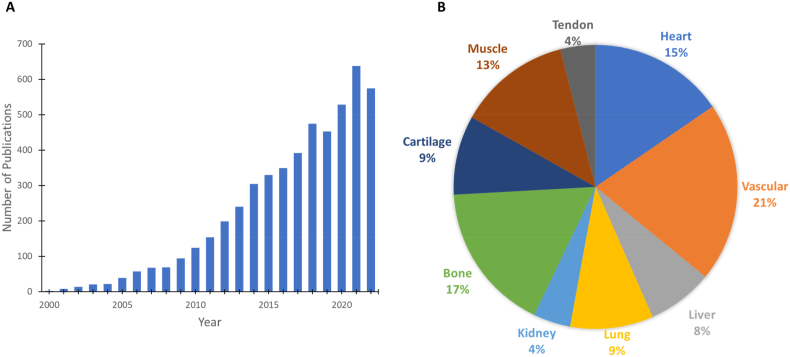
Decellularized tissue biomaterials/grafts. (A) SciFinder published article results for “Decellularized Tissue” by year since 2000, showing exponential increase in research interest and progression over the years. (B) Break-down of the articles published according to tissue/whole organ application. Heart, vascular and bone tissue engineering are the top three areas where dECM is used as a biomaterial or graft. We used a total of 5386 published articles to develop the presented pie chart.

Decellularized tissue has recently gained considerable notice as a potential biological scaffold. The use of decellularized tissue involves processing for the removal of cellular components whilst preserving/minimizing loss of tissue- and/or organ-specific ECM properties and in some cases, the preservation of vascular and neural networks and architecture. The maintenance of the intrinsic biochemical and biophysical cues of the native ECM provides structural and chemical signaling cues for regulating cellular behavior in terms of supporting cellular adhesion, migration, proliferation and differentiation [23]. These include biochemical cues such as structural proteins, peptides and cytokines/GFs as well as biophysical and material properties such as 3D structure, porosity and mechanical properties similar to native tissue. Additionally, decellularized ECM (dECM) also contains an abundant supply of tissue-specific GFs and other signaling molecules [24,25]. Together these play an important role in the development of cellular microenvironment niche and holds great promise for decellularized tissue biomaterials as natural bio-instructive materials/grafts to regulate and facilitate tissue-specific cellular behavior and stimulate regeneration. As such, there has been an exponential growth in research interest and progression with the use of decellularized biomaterials/grafts since 2000s (Fig. 1A).

The development of dECM for use as natural instructive scaffolds and their functional outcome involves efficient decellularization of the donor tissue which can later be re-seeded with various types of cells. Simple tissues, such as bone, cartilage, muscle, tendon, vascular, and complex organs, such as heart, liver, lung, kidney are decellularized for tissue engineering and regenerative medicine applications (Fig. 1B) [[26], [27], [28], [29], [30], [31], [32], [33], [34], [35], [36], [37], [38], [39], [40], [41], [42], [43], [44]]. A number of reviews on this topic covered decellularized biomaterials in various forms (hydrogels, bio-inks, and organized porous structures) and their biological, and medical engineering applications including disease modeling and drug screening platforms [31,[45], [46], [47], [48], [49], [50], [51], [52], [53], [54]].

This comprehensive review focuses on decellularized tissue-/organ-derived biomaterials and cell-derived biomaterials as an emerging frontier in tissue engineering and regenerative medicine. We identify the decellularization effects on mechanical as well as biological properties and discuss mitigation techniques with the goal of mimicking native tissue and organ structure as well as biological function. We discuss the need for sterilization and preservation methods of dECM products that are often ignored but have clinical prominence to meet GMP standards. A special attention is given to the emerging area of research that explores the chemotactic ability of the decellularized tissue/organ and their use in developing *in situ* tissue regeneration strategies. The review also highlights recent trends and developments, contemporary challenges, and clinical prospects of the dECM biomaterials and grafts.

## 2 Decellularization methods

Allogenic/xenogeneic sourced tissue-/organ-derived materials have been widely studied for use in tissue repair and organ transplantation strategies [23]. However, the presence of foreign cellular material and antigens such as alpha-gal epitope can lead to adverse cell/host responses leading to rejection and thus requires the minimization/elimination of immunogenic risk before use as a biomaterial or graft for TE [55]. For that reason, numerous methods have been developed for tissue/organ decellularization for the removal of cellular and genetic material. The goal of the decellularization process is to completely remove cellular material while preserving ECM structure and bio-chemical composition. The three classifications of common methods of decellularization include: physical, chemical and enzymatic methods. Tissue or organ decellularization methods rely on immersion or perfusion of solutions containing chemical or biological agents, or physical stresses for cellular membrane disruption, to induce cell death and removal. The choice of decellularization agents, duration of treatment and protocol involvement/complexity is dependent on the target tissue/organ properties such as the tissue thickness, density, structure, origin and lipid content along with its intended use [19]. Commonly used tissue/organ decellularization, sterilization and preservation methods, along with their effects on ECM are shown in Table 1 and Table 2.

**Table 1:** Physical, Enzymatic, and Chemical Methods commonly applied to decellularize tissues and organs.

Category	Treatment/Technique	Effect on Tissue	Ref.
*Decellularization Method*			
**Chemical**			
	Acids & Bases	-Causes/catalyzes hydrolytic degradation of biomolecules and solubilizing cytoplasmic components	[252]
	Hypotonic/Hypertonic Solutions	-Causes cell lysis due to induced osmotic effects -Most often combined with other decellularized agents	[253,254]
**Detergents**		-Destroys lipid-lipid & lipid-protein interactions, solubilizing plasma membranes of cells and dissociating genetic material from proteins	
	Non-ionic Detergents (e.g. Triton X-100)	-Non-denaturing detergent that causes cell lysis and removal of cellular residues -Limited in ability to break protein-protein bonds	[254,255]
	Ionic Detergents (e.g. SDS)	-Modifies protein structures due to charge difference -Disrupts cell membranes	[256,257]
	Zwitterionic Detergents (e.g. CHAPS)	-Non-denaturing detergent with properties of both ionic and non-ionic detergents -Milder agents with greater ECM structural maintenance -Incomplete cytoplasmic removal	[118]
**Enzymatic**			
	Nucleases (DNase, RNase, etc.)	-Catalyzes the cleavage of phosphodiester bonds of nucleotides, fragmenting DNA and RNA for inactivation, prevention of replication and elimination	[258]
	Trypsin	-Cleaves peptide bonds of arginine and lysine -Commonly used in combination with chelating agents such as ethylenediaminetetraacetic acid (EDTA) used to bind to divalent cations at cell-adhesion sites to cause dissociation	[259,260]
	Lipase	-Catalyzes the cleavage of ester bonds in lipids	[261,262]
	Dispase	-Protease that mainly cleaves fibronectin and collagen IV proteins	[263,264]
	Collagenase	-Cleaves peptide bonds in collagen	[265,266]
**Physical**			
	Freeze-Thaw Cycling	-Can consist of one or more freeze-thaw cycles -Lyses cell membrane via ice crystal formation -Range of temperature −80 °C – 37 °C	[[267], [268], [269], [270], [271]]
	High Hydrostatic Pressure	-Relatively quick decellularization process -High pressure disrupts cell membrane inside tissue	[[272], [273], [274], [275], [276]]

**Table 2:** Decellularization Tissue Sterilization and Preservation methods for developing dECM biomaterials/grafts for clinical use.

Category	Treatment/Technique	Effect on Tissue	Ref.
*Sterilization Methods*			
	Gamma and Electron irradiation	-Radiation based destruction of existing microbes -Destruction of DNA and prevention of microorganism replication, however, can cause damage to ECM collagen network	[277,278]
	Ethylene oxide (EtO)	-Irreversible alkylating agent -Prevents replication of microorganisms by damaging DNA -Suppresses cellular metabolism and division and inactivates many bacteria and viruses	[58,60]
	Supercritical carbon dioxide (scCO_2)_	-Penetrates ECM and inactivates microorganisms present with minimal structural disruption - Relatively non-toxic, attractive method for the creation of constructs with high biocompatibility	[279,280]
*Preservation Methods*			
	Lyophilization	-Vacuum sublimation process for water removal through sublimation of ice -Protein stabilization at room temperature for long term storage - Required subsequent reconstitution step	[65,262]
	Cryopreservation	-Slow-rate freezing or snap freezing -Requires use of cryoprotective agents to mitigate damaging effects of ice crystal formation - Stabilizes material for long term storage by preventing degradation of biological molecules	[281]
	Antibiotics and Antimycotics in −20/-80 °C	-Inactivates bacteria through specific intracellular targeting and destruction - Slows chemical processes and degradation for short-long term storage	[282]

Chemical reagents are commonly employed for the solubilization of cellular membranes, dissociation of DNA and disruption of lipid-protein interactions. Chemical reagents for decellularization include acids and bases, hypo/hypertonic solutions and detergents (ionic/non-ionic). Unlike chemical reagents, the implementation of enzymatic methods to decellularize tissue allows for the removal of undesirable components and cellular residue with high specificity for biological molecules. Enzymes are commonly implemented alongside other decellularization methods to accentuate cellular and genetic material removal by cleavage of cell-cell/ECM interactions and nucleic acids. Deleterious effects towards critical ECM components and substantial toxicity of commonly used chemical and enzymatic decellularization methods have incentivized the potential efficacy of other methods utilizing physical modalities to be investigated. Physical methods can prevent disruption of ECM structure, however, are typically ineffective for efficient decellularization on their own. It is commonly seen for physical methods to be combined with chemical and/or enzymatic modalities to accentuate the removal of cellular and genetic material by improving the penetration of other decellularization agents [56]. The improvement of decellularization agent infiltration is paramount for avascular tissues, such as hyaline cartilage and fibrocartilage. Additionally, all physical methods require the rinsing of the tissue to flush the structure of cellular and genetic material present after the physical method is implemented. Physical methods for decellularization include the use of freeze-thaw cycling, and high hydrostatic pressure.

## 3 Decellularized tissue preparation for medical use

### 3.1 Sterilization

Preceding implantation, it is paramount that biological constructs are sterilized and rid of existing genetic material and residual bacterial and viral content to minimize immunogenicity risk [57]. Commonly implemented terminal sterilization techniques include utilization of gamma or electron beam radiation, ethylene oxide and supercritical CO_2_. In addition, antibiotics/antimycotics are routinely used sterilization techniques during decellularization and handling in aseptic conditions. Moreover, the method of choice is dependent on size and complexity of the decellularized tissue graft and must prevent structural damage and ECM changes. Gamma irradiation (GI) is a cold process where the temperature of the sterilized product does not increase substantially, thus making it a suitable option for sterilizing biologically relevant materials. The source used for the sterilization process is the radioactive isotope, cobalt-60 [58]. Due to the insufficient energy, products treated with gamma irradiation via the cobalt-60 isotope do not become radioactive, resulting only in the destruction of existing microbes [59]. Electron irradiation (EI) is another cold sterilization process that utilizes radiation. Conversely, EI uses an electron accelerator as a source for its radiation. Both processes damage DNA and thus prevent replication of microorganisms that exist in the graft, however, can cause damage to the ECM collagen network. Processing materials with ethylene oxide (EtO) involves the exposure of the material to ethylene oxide gas. EtO acts as an alkylating agent that prevents the replication of microorganisms by damaging DNA and prevents cellular metabolism and division [60]. EtO is limited in terms of how much it penetrates the material, thus it only affects the surface. As well as being used as a means to decellularize tissue, supercritical CO_2_ (scCO_2_) has also been observed to have a sterilizing effect and has been used to sterilize natural [61] and synthetic [62] biomaterials. Due to the low viscosity and high diffusion coefficients, scCO_2_ liquid is able to penetrate biological grafts and extract undesirable material without causing substantial disruption of structural integrity and mechanical performance of the tissue [61]. This benefit is accentuated in thick tissues that require adequate penetration to decellularize and sterilize effectively. Moreover, the implementation of scCO_2_ is relatively non-toxic, making it an attractive decellularization/sterilization method for producing a construct with no immunogenicity [63].

### 3.2 Preservation

In addition to sterilization of final decellularized tissue constructs, preservation is also an important step as freshly decellularized constructs are often not feasible due to shortage of supply, requiring long-term preservation techniques. The goal of preservation is to have on-shelf products for clinical use. Considerations must be taken for developing appropriate preservation techniques for quality assurance and clinical translation of these tissue-derived products. Commonly implemented preservation techniques include lyophilization, cryopreservation and utilization of anitbioitics and antimycotics stored at −20 °C/-80 °C. For long-term storage of decellularized tissue grafts, they may be lyophilized to better preserve the material without causing substantial damage to the construct during processing. In order to prevent the formation of large ice-crystals, which may cause physical damage to tissues, low pressure is used to hasten the freezing process. By being performed at low pressures, the majority of the water present in the material is sublimated. Proceeding from the sublimation phase, the temperature is raised to break bonds between water ionically bounded to the construct, further drying the construct. This method is performed with non-toxic protective agents and eliminates need of low temperature storage [64]. Moreover, cryopreservation which involves the use of 10% DMSO and slow-rate of freezing or snap freezing in liquid nitrogen has also led to the preservation of tissue grafts with histological resemblance to non-preserved grafts [65]. Other methods of long-term preservation include the maintenance of decellularized tissue constructs in PBS containing antibiotics and antimycotics and stored at 4 °C, however only suitable for short-term preservation as well as stored at − 20 °C and −80 °C for longer term preservation [66,67]. Nevertheless, current methods lead to a progressive degradation of tissue architecture with compromising biomechanical properties limits clinical applicability and warrants further investigations [68,69].

## 4 Decellularized ECM-based grafts: various forms

Similar to the plurality of possible decellularization methods, there are also many possible graft forms that can be prepared from decellularized tissue. Common varieties include so-called “2D” scaffolds, ECM powders, hydrogels, composite grafts, and whole organs. Physical and chemical properties vary between graft types - the desired application dictates which type of graft form is used.

Clinical success has been shown for more “simple” graft architectures such as skin grafts and vessels, while complex grafts such as whole organs remain a challenge [70]. In clinical settings, intact tissue remains the most commonly used variety of decellularized graft tissue. Intact tissue refers to a graft that maintains its geometry once decellularized (it is not turned into a powder or hydrogel). The most popular clinically available intact grafts are decellularized dermis products such as GraftJacket®, Integra®, Dermagraft®, Apligraft® and Allopatch®. These decellularized dermis grafts are largely composed of collagen, making them versatile skin grafts. Intact decellularized dermis grafts are also used in tendon repairs, bone regeneration, hernia repair, wound healing etc.

### 4.1 Intact/powder ECM

Decellularization often compromises mechanical properties of tissues depending on the decellularization protocol employed; consequently, it is critical that most of the native ECM is not disrupted or removed while processing. Post-decellularization, tissues can be used for tissue remodeling and regeneration as grafts provided the ECM retains its functionality and provides necessary cues to support cellular proliferation and differentiation for guiding *de novo* tissue formation.

In a study using a decellularized vein as a graft for vascular tissue engineering, strategies were used to decellularize human greater saphenous veins, and its structure and composition were evaluated. Ultimately, the scaffold should retain enough of its native ECM for strength and stiff environment for cell attachment. Vascular application of this graft requires maintenance of structural and mechanical integrity to endure arterial implantation for its initial strength and pressure that occurs post-implantation [71]. The vein was prepared using a chemical detergent SDS to remove cells and washed with PBS. *In vitro* mechanical integrity assessment showed insignificant alterations to burst and suture strength given the decellularization processing; as a result, strengths were similar to that of the fresh vein. In applications demanding structure and mechanical integrity, intact decellularized tissue ECM is preferred over further processed tissue/organ ECM.

While intact tissues provide original tissue vasculature, the shape and form of the original tissue, and retained mechanical strength, decellularized tissue processed as a powder offers benefits that intact tissue cannot. Fabrication of tissue ECM powder consists of freezing and lyophilization of the decellularized tissue followed by pulverization and milling [72]. Additionally, powder can be formed by snap freezing the lyophilized tissue and grinding the product in a mill. The sample endures temperatures below −70 °C by using liquid nitrogen for the purpose of preserving the tissue. Prior to snap freezing, samples can also be saturated in NaCl to promote salt crystal precipitation; as a result, the powder shows a more uniform distribution once ground. Powdered tissue can fill areas and mold with the shape of the tissue defect; its ability to conform allows for minimally invasive procedures for implantation. ECM powders offer versatility in that they are often converted into gels and inks to be used as injectables. When constructing these powders and considering their applications, special attention is given to particle size, powder solubilization, and ECM crosslinking. For example, a range of particle sizes in a powder will still allow for cell growth in applications, but providing uniformly sized and distributed particles promotes homogeneous tissue formation. Attention to powder solubilization is also critical if the application requires processing these powders into injectable hydrogels. The derived powder is generally solubilized using hydrochloric acid to ensure proper enzymatic digestion. For *in vivo* use, the acidic pH from solubilization must be neutralized to a pH naturally found in the area of implantation. Because processing decellularized tissues into powders compromises its mechanical integrity, ECM protein crosslinking is usually performed to account for the lost strength.

Decellularized tissue powders were applied in an approach to create “tissue papers” using dECM powders obtained from porcine and bovine tissues and organs [73]. The powder was prepared through a variety of processes including dicing, decellularizing, washing, lyophilizing, and mechanically milling tissue. The use of this dECM powder allowed for control of the desired “tissue papers” as the powdered suspension was converted into an ink to be poured and dried for maximum control of size and shape. The conversion of powder to ink consisted of introducing the powder to a solvent mixture including evaporants, surfactants, plasticizers, and solubilized PLGA. Because mechanical integrity can be lost as a disadvantage in creating dECM powders, the ECM was mechanically milled into approximately 200 μm particles which showed to be large enough to retain mechanical properties in its native structure. *In vitro* results showed the adhesion, viability, and proliferation of human MSCs on “tissue papers” derived from decellularized porcine heart, kidney, liver, muscle, bovine ovary, and uterus.

In another study, Mazzitelli et al. utilized a powdered form of a decellularized urinary bladder to co-exist with Sertoli cells, or epithelial cells of testes, encapsulated within alginate based microparticles. This study was designed to investigate the effect of urinary bladder matrix (UBM) decellularized powder on the morphology of the alginate based microparticles, cell viability and behavior of Sertoli cells [74]. The incorporation of UBM in powder form was used to increase cell survival and function. Preparing UBM powder consisted of decellularizing porcine urinary bladder, which was prepared in sheets and then lyophilized, and mechanically milled into particles. Results showed increased levels of laminin, and integrin expressions for Sertoli cells encapsulated in UBM powder.

### 4.2 Decellularized tissue-derived hydrogels

Decellularized ECM tissue powder can be further solubilized and manipulated to form hydrogels. Decellularized tissue-derived hydrogels have expanded the potential use of dECM *in vitro* and *in vivo* as culture substrates that are both injectable and 3D printable [75,76]. This allows for their use to fill irregularly-shaped defects in a minimally invasive manner and to create precisely controlled decellularized tissue grafts [77].

The formation of hydrogels derived from decellularized tissue/organ typically involves the pepsin-solubilization of the dECM followed by physical crosslinking or the self-assembly of the collagen fibers to form a 3D network [78]. Smooth muscle ECM from caprine esophageal tissue was decellularized using hypo and hyper-molar sodium chloride solutions alternatingly, solubilized and then constructed into a hydrogel after adjusted to physiological pH and temperature [76]. The detergent-free method of decellularization improved the retention of sGAG and collagen content and ECM constituents. Moreover, the study demonstrated that the hydrogel induced differentiation of encapsulated adipose-derived mesenchymal stem cells towards smooth muscle cells (SMCs) without externally added factors, through expression of alpha-smooth muscle actin and myosin heavy chain, the hallmarks of SMCs. ECM-derived hydrogels are also utilized to deliver soluble factors, such as growth factors/biologics, and improve retention of transplanted cells [79]. Wu et al. utilized decellularized cardiac muscle tissue which self-assembled into a nanofibrous hydrogel at physiological temperature and was used to encapsulate cardiomyocytes (CMs) and loaded with SDF-1α [75]. The hydrogel improved retention of transplanted CMs and the GF promoted recruitment of endogenous cells. Intramyocardial injection of the hydrogel solution to the infarcted area led to the promotion of angiogenesis, inhibition of fibrosis, reduced infraction size and improved cardiac function. ECM-based hydrogels have the ability to mimic the physiological matrix environment, promote cellular adhesion, infiltration and proliferation. However, they often exhibit poor mechanical strength, rapid degradation and poor stability [80]. In efforts to mitigate these, researchers developed graded-concentration hydrogels composed of porcine urinary bladder matrix (UBM) as a dermal scaffold for chronic wound treatment [81]. Hydrogels were designed as a three-tiered gradient hydrogel of different concentration (low and high). The gradient dUBM hydrogel showed stability of cross-sectional area during collagenase degradation despite considerable loss of mass, as well as resisted fibroblast mediated contraction while supporting high surface cell viability through mechanical support provided by the denser layers of the dUBM.

**Fig. 2:**
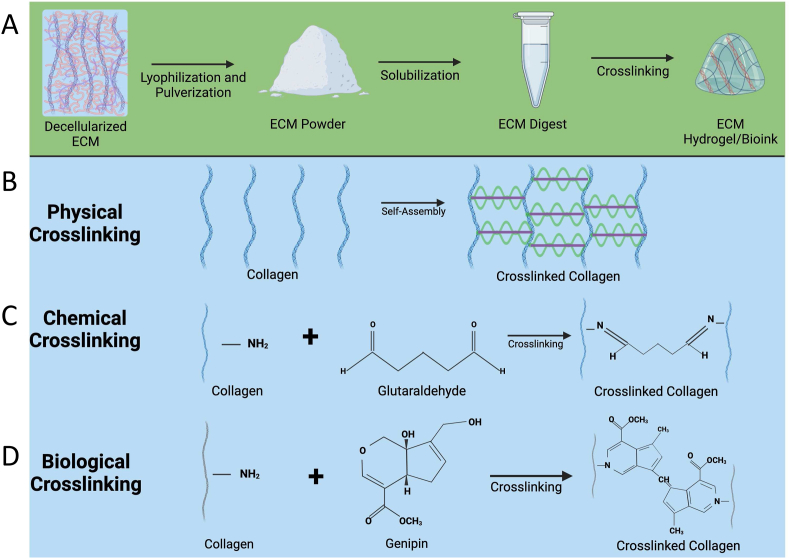
Development of dECM hydrogels/bio-inks. In the schematic representation (A), decellularized tissue undergoes a series of processing steps to form hydrogels/bio-inks. Initially, the tissue is decellularized, followed by conversion into a powder form. Subsequently, the powder is solubilized and digested to obtain dECM hydrogel. Cross-linking of dECM is essential to maintain mechanical strength and the stability of the structure in a biological environment. Different crosslinking methods can be employed for dECM cross-linking, as illustrated in panels (B), (C), and (D). Physical crosslinking (B) involves self-assembly under physiological conditions. Chemical crosslinking (C) can be achieved using agents like Glutaraldehyde. Biological/enzymatic crosslinking (D) can be facilitated using biological agents or naturally available materials such as genipin.

The self-assembly of hydrogels allow for milder crosslinking with improved cytocompatibility, however, these hydrogels are often mechanically weaker, have decreased stability and undergo rapid degradation, hindering their applications for tissue engineering. To improve these properties, hydrogels are often modified and subjected to chemical or biological crosslinking methods, as depicted in Fig. 2. In one study, human bone fragments were demineralized, decellularized and further processed by functionalizing with methacrylate groups to form a photocrosslinkable methacrylate bone ECM hydrogel [82,83]. The mechanical properties of the hydrogel demonstrated tunable mechanical strength with elastic modulus increasing as a function of photocrosslinking time while still retaining the nanoscale feature of the polymer networks. The resulting hydrogel demonstrated high cytocompatibility that supports vascularization of endothelial cells and led to the formation of an interconnected vascular network likely due to the presence of pro-angiogenic biomolecules in the bone ECM. Polyethylene glycol diacrylate (PEGDA) and decellularized annulus fibrosus matrix (DAFM) was combined to develop an injectable photocurable hydrogel for annulus fibrous repair [84]. The addition of PEGDA improved the mechanical strength of the DAFM hydrogels whilst maintaining the porous structure. The hydrogels were loaded with TGF-β1 and *in vivo* repair performance was assessed using a rat annulus fibrosus (AF) defect model. The implantation of the hydrogel sealed the AF defect, prevented nucleus pulposus atrophy, retained disc height and partially restored the disc biomechanical properties. Other chemical/covalent crosslinking agents, such as glutaraldehyde (GA), and carbodiimides or the use of secondary components that are capable of chemical crosslinking can be used to develop dECM biomaterials and grafts with the desired physio-chemical characteristics [84,85]. Compared to physical crosslinkers, chemical crosslinking creates more stable hydrogels, however there are concerns of possible cytotoxicity. The large volume of hydrogel biomaterial data that is available may help us to determine the safe chemical-crosslinkers to develop relatively stable and functional dECM biomaterials and grafts [86,87].

Since dECM is a tissue-derived material, researchers have also looked at using biological crosslinking methods. Common biological crosslinking agents include genipin and transglutaminase (TG) [86,88]. Genipin is derived from gardenia fruit and can bind with free amine groups of lysine or hydroxylysine to crosslink collagen hydrogels. Biological crosslinkers are naturally available chemical agents that can create cross-links and form covalent bonds with dECM. Due to their biological origin, they are proven to show improved biocompatibility. Genipin crosslinked decellularized nucleus pulposus hydrogels were developed and evaluated in a rat degenerated coccygeal intervertebral disc model [89]. The hydrogel developed with optimal genipin concentration (0.02%) demonstrated similar elastic modulus to human nucleus pulposus (NP), good biocompatibility and inducibility of expressing NP-related genes. *In vivo* studies showed that the hydrogel supported the survival of adipose-derived mesenchymal stem cells, improved the intervertebral height, and histological grading score. In another study, genipin-terminated 4 arm-poly(ethylene glycol) (GeniPEG) was synthesized. dECM-based hydrogels were formed by mixing GeniPEG and dECM at an optimum pH through crosslinking of dECM and self-crosslinking between the GeniPEG molecules [90]. The hydrogels crosslinked with GeniPEG exhibited greater tissue adhesive strength to porcine-derived aorta tissue compared to genipin crosslinking. *In vivo* studies demonstrated biocompatibility and biodegradability of the hydrogel which later expanded to be used for dECMs-GeniPEG hydrogels for sealing wounds and preventing post-operative complications.

In a systematic study, physical, chemical and enzymatic crosslinking methods were compared in developing dECM hydrogels [87]. Hydrogel derived from human umbilical cord were subjected to genipin or EDC crosslinking and the mechanical, degradation stability and biocompatibility were evaluated. Genipin and EDC crosslinking slowed the gelation time and increased the resistance against *in vitro* enzymatic degradation compared to physically crosslinked hydrogels, with genipin being more effective. Genipin crosslinking also revealed improved rheological properties compared to physical crosslinking. Both genipin and EDC crosslinking also enhanced the bio-stability without affecting mesenchymal stem cell proliferation, and neural stem cell growth and differentiation. *In vivo* studies demonstrated that genipin crosslinking allowed for *in situ* gelation and improved ECM retention for up to 2 weeks without any adverse tissue response or enhanced inflammatory reaction. These studies suggest that decellularized tissue can be modified via physical, chemical, or enzymatic cross-linking based on the need. For instance, temperature induced cross-linking (hydrogen bonding/collagen self-assembly) is used to form decellularized tissue gels for short-term or *in vitro* evaluation studies. On the other hand, dECM can be modified with chemical/biological cross-linking agent (covalent bonding) to develop decellularized tissue biomaterials/grafts for tissue repair and regeneration studies.

### 4.3 Composite grafts

Tissue-derived grafts are often used in the composite form to address loss of mechanical strength or inadequate mechanical behavior of the produced grafts. Additionally, tissue engineered grafts are designed to provide an environment in which cell-cell interactions as well as the surrounding matrix in terms of the bio-chemical composition can be controlled for a desired cell behavior and performance.

Of materials that are incorporated to assist the decellularized tissue, synthetic and natural polymers show promising features allowing for enhanced mechanical properties, desirable porosity, controlled degradation rates, and increasing binding sites. Producing polymer fibers acts to mimic the fibrous nature of the tissue ECM. Accurately creating these synthetic polymer fibers is done through methods such as electrospinning where critical characteristics such as fiber diameter and orientation can be completely controlled. The need for composite scaffolds arises in situations such as thrombus formation in the vasculature of decellularized tissues. Because the ECM is highly thrombogenic, it is critical that ECM in the engineered graft is not exposed to blood. In a study to address this, a polyester elastomer, poly (1,8 octanediol citrate) (POC) was incorporated to link heparin to the ECM based scaffold; as a result, the increased interaction of heparin and the ECM allowed for a decrease in thrombosis [77]. It was also seen that the presence of heparin immobilization after incorporating POC within the decellularized tissue, lead to increased proliferation of HUVECs which in a way moderated the interaction between ECM proteins and blood. In addition to cell proliferation and heparin binding, POC also offers mechanical integrity to the vasculature of the tissue thus providing mechanical support in heart valve tissue engineering. As decellularization removed some ECM components along with cells from the porcine aortic valve, the mechanical characteristics of the valve were also compromised. In efforts to address this, biodegradable polymers such as polyhydroxybutyrate (P3HB) and poly-4-hydroxybutyrate (P4HB) were introduced to a dehydrated decellularized tissue followed by rehydration. The addition of polymers led to increased biomechanics in suture retention and tensile tissue strengths of the dECM constructs [78]. Results showed increased proliferation of mouse fibroblasts on the composite graft *in vitro* using dECM-polymer composite grafts. *In vivo* studies conducted using a rabbit abdominal aorta patch implantation model, resulted in early inflammatory cell infiltration which steadily reduced in the following weeks. Also, there was no calcification or wall thickening present that would compromise the vessel's functionality.

Although a degree of degradation is desirable for the composite grafts, it is crucial that the integrity of the graft is not compromised during both decellularization and recellularization. For the best results, degradation rates of grafts should be at a slow enough rate where it can maintain cell proliferation while also eventually allowing for the production of the new ECM to create *de novo* tissue. In a study, three polymer compositions of different degrees of degradation were used to test the composite grafts for mechanical performance and degradation characteristics. For this reason, different structural compositions of polymers were used to determine a proper degradation rate. Polymers with high degradation rates lost a significant amount of mechanical integrity and became very brittle by the end of the cellularization-decell-recell cycle. It was found that the polymer with medium degradation rate allowed for the best ECM deposition while also maintaining the structure of the ECM. Polymers can also be incorporated as fiber mats through electrospinning tyrosine-derived polycarbonates. Another study utilizing different extracellular matrices with polymeric structures showed that chondrocytes cultured in these fiber mats displayed higher differentiation tendencies. The pDTEC fiber mat in the form of template structure provided mechanical basis and stiffness for *in vitro* testing of cell proliferation and differentiation [91]. When introduced, ECM covered the fiber mat surface while also retaining pore structure leading to increased cell attachment.

There are several advantages of incorporating synthetic and natural polymers in ECM grafts including their biocompatibility, biofunctionality, and having control over physio-chemical properties. Composite scaffolds also allow for the incorporation of tethered or immobilized peptides and GFs to further enhance bioactivity and performance of dECM. In addition to incorporating polymeric fibers, osteoinductive components such as hydroxyapatites and calcium phosphates can be introduced to synergistically improve osteogenic performance of the composite grafts for bone tissue engineering. In a study where chitosan and nano-hydroxyapatite particles were incorporated onto a decellularized goat-lung matrix, factors such as osteoblast attachment and proliferation were enhanced. The increased degree of crosslinking between the collagen-based tissue and CS/nHAp composite allowed for better stiffness and attachment of cells. The incorporation of the CS/nHAp provided a bone-like environment which led to enhanced cellular attachment and increased proliferation of the seeded osteoblasts. Not only did the additive CS/nHAp provide the desired environment for cells, the resulting surface roughness on the composite ECM graft also increased the tendency for cell attachment.

### 4.4 Whole organ

Whole organ decellularization serves as a method to provide an organ template to be recellularized and implanted in order to address donor organ shortage. When preparing for whole-organ decellularization, it is critical to consider the resulting decellularized organ composition and potential host response post-implantation. In addition, the vascular and neural network of the decellularized organ to be intact to allow for re-cellularization.

Antegrade or retrograde perfusion is a common method to decellularize whole organs without disrupting the organ's structure [92,93]. This technique delivers decellularizing agents through the vasculature of the organ. Retrograde perfusion has been performed on hearts where agents such as Triton X-100 and SDS are delivered through a cannulated aorta followed by perfusion of deionized water and PBS. Perfusion was again performed to ensure that the vasculature of the heart remained intact after delivering decellularizing agents. An advantage to decellularization by perfusion is the ability to control the time of decellularization by adjusting the pressure at which the perfusate is pumped. Progressively increasing the perfusion pressure causes the vessels to dilate; as a result, flow rate increases allowing for quicker cell removal. Because of the progressive increase of pressure, the vessels are not damaged from the dilation.

In addition to hearts, perfusion is also an advantageous technique in decellularizing lungs as they contain two systems of entry for perfusion: the vasculature and airway and alveolar system. Because of the additional structure of the airway compartment, decellularization perfusion can be adequately performed strictly through the vasculature as it is done with hearts, or through the airway compartment and alveolar structures to quicken the process. To further remove cellular content, organs can be repeatedly incubated with NaCl and DNase to discard any remaining nuclei and DNA [94]. Another way to remove cell residue is distributing supercritical carbon dioxide through the tissue. This process is commonly used as it provides an inert gas and allows for the maintenance of the organ basic structure and mechanical integrity.

To tackle liver disease and shortage of donor organs, Uygun et al. presented a potential solution in the form of generating decellularized livers for transplantation [95]. The rat livers were decellularized through portal vein perfusion with special attention given to maintaining the vascular network, which would be reconnected to the body's circulation after implantation. To ensure that the vasculature remained intact, Allura Red dye was delivered through perfusion, which clearly displayed the vascular network within the translucent matrix. Although the study displayed an efficient decellularization process for rat livers, some of the vascular integrity was lost due to the removal of nonparenchymal cells, such as the liver sinusoidal endothelium. As a result, the recellularization process could be improved to incorporate nonparenchymal cells to restore vascular network and their integration with the body's circulatory system.

## 5 Decellularized ECM: tissue engineering applications

Developments in techniques for decellularized ECM-based biological scaffolds have come a long way and have since been increasingly considered for tissue engineering and regenerative medicine strategies [92]. Initial and current advancements in whole organ decellularization focus on perfusion-based techniques, taking advantage of the large native vascular network. Perfusion decellularization is based on the pressure induced perfusion of various detergents, chemicals and enzymatic treatment through the vasculature network for the removal of cellular material with minimal damage to vital ECM components and 3D architecture. Perfusion based decellularization treatments largely depend on the mechanical, thickness/density characteristics and type of the whole organ. For instance, complex organs often require higher concentrations and longer exposure times of agents for complete decellularization without compromising the native ECM. The acellular organ constructs are then recellularized with relevant autologous or stem cells and cultured in a bioreactor for the development of functional tissue engineered organs to alleviate the shortage of available organs for transplantation. Many organs have been decellularized for this purpose including heart, liver, lung, and kidney.

### 5.1 Heart

In 2008, Ott et al. first described decellularization of rat hearts through coronary perfusion of four different detergents [96]. The resultant decellularized tissue was visibly translucent, allowing for reperfusion following treatment. Upon recellularization with neonatal cardiomyocytes, heart tissue showed electric and contractile responses to single pace electrical stimulation. In efforts to optimize decellularization methods for preservation of ECM properties, Seo et al. developed a detergent free method using supercritical carbon dioxide and ethanol co-solvent treatment [97]. Decellularized heart tissue retained more ECM components such as collagen, GAGs, laminin, fibronectin and angiogenic factors, compared with the detergent treated tissue.

Several recent studies have also focused on developing decellularized heart valves with anti-calcification properties. Efforts include the formation of a balanced charged network that prevent the transport of Ca^2+^ ions and enzymes, use of VEGF encapsulated within PCL nanoparticles and the use of osteoprotegerin [[98], [99], [100], [101]]. In order to enhance *in vitro* recellularization of heart valves, VeDepo et al. investigated the effects of bioreactor conditioning parameters [102]. Reseeded ovine aortic heart valves were exposed to varying conditions of hypoxia/normoxia and high/negative cyclic pressures. Results demonstrated that hypoxic conditioning led to increased cellular infiltration into the valve leaflet tissue compared to normoxic conditioning. In another study, a porous MMP degradable PEG hydrogel incorporated with SDF-1α combined with decellularized porcine aortic valves were fabricated [103]. The hydrogel inclusion led to enhanced BMSC adhesion, viability and proliferation, as well promoted MSC recruitment while facilitating M2 macrophage phenotype polarization in a rat subdermal model.

To overcome the major obstacles in the field, research has been focused on the development of cardiac patches with functional vascularization for the repair of malformed/damaged myocardium and valve reconstruction [[104], [105], [106]]. In one study Jang et al. developed a hdECM-based bio-ink that was 3D printed for fabrication of multiple cell-laden patches (cardiac progenitor cells [CPC] and MSCs) [105]. The precise dual patterning of the cells and use of tissue-specific bio-ink with VEGFs promoted vascularization with enhanced cardiac function when implanted subcutaneously in nude mice, compared with a CPC patch. In another study, decellularized myocardium slices (dPMS) were reseeded with hMSCs with the goal of assembling prevascularized tissue. These dPMS supported cell attachment and survival, demonstrated thickness dependent cell seeding efficiency, and induced endothelial differentiation of hMSCs. This study noted limitations with static seeding and efforts have been made by other researchers using perfusion bioreactors to enhance recellularization cell density and subsequent vasculature formation of thick constructs [107].

### 5.2 Liver

Perfusion based decellularization and recellularization via the vasculature of the construct has also been explored for other organs with differences in vasculature mode of entry/routes available for decellularization and redelivery of cells for functional repair. Liver decellularization has been performed through perfusion via the portal vein, inferior and superior vena cava or the hepatic artery [108]. In 2010, Uygun et al. first utilized SDS for the decellularization of liver tissue via the portal vein over the span of 72 h [95]. Immunological staining revealed retention of ECM proteins namely collagen type I and IV, fibronectin and laminin-β1 and approximately ∼50% of GAGs.

Efforts in the recent years have been to accelerate and improve the efficacy of the decellularization procedure [[109], [110], [111], [112]]. In a study by Willemse et al., porcine livers were decellularized using Triton X-100 or Triton X-100 in combinations with SDS, maintained under constant pressure perfusion (120 mm Hg) [113]. Analysis post decellularization revealed effective cell and DNA removal with the Triton X-100 only protocol retaining 1.5 and 2.5 times more collagen and sGAG, respectively compared with treatment with Triton X-100 and SDS. When applied to human livers, the Triton X-100 only decellularization protocol with pressure-controlled perfusion showed semi-transparent liver within 20 h, which was otherwise not achieved without pressure-controlled perfusion within a span of 64–96 h. Another study decellularized whole porcine livers using milder agents, namely through perfusion of saponin, sodium deoxycholate and deionized water. Using this method, authors produced an acellular scaffold with intact vasculature and preserved ECM (including collagen I and IV and laminin) in less than 24 h, reducing the time even further [108]. A study by Watanabe et al. sought the formation of hierarchical vascular network in recellularized livers to overcome the challenge of damaged vascular network during decellularization [114]. By subjecting fibronectin coated decellularized livers to perfusion culture at 4.7 ml/min, authors demonstrated the formation of sinusoid-scale micro-vessels via angiogenesis, which was otherwise not observed in static culture. These studies suggest that mechanical and adhesion factors may play a role in construct of vascular networks. Sustained perfusion for up to 15 days has also been recently demonstrated in immunosuppressed pigs [115]. The recellularization of livers and the re-establishment of the biliary system and vasculature are of utmost importance for the restoration of bile flow and proper hepatic function.

**Fig. 3:**
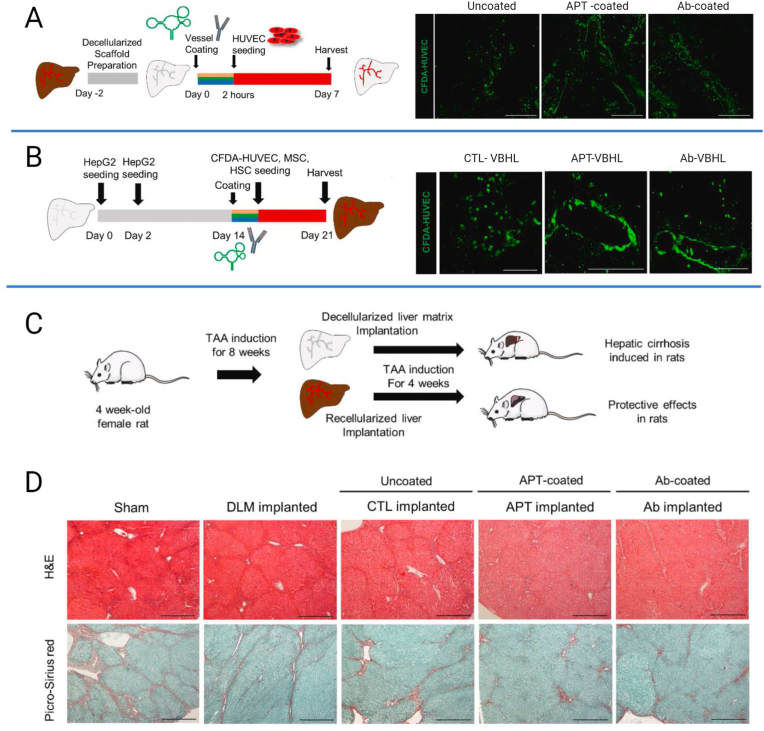
Generation of VBHL constructs for vascularized liver reconstruction. (A) Schematic diagram illustrating the strategy for re-endothelialization of the decellularized rat liver constructs with HUVECs. Representative immunofluorescence images of endothelialized vessels with CFDA-labeled HUVECs in the scaffolds without the coating agent (Uncoated) or with the following coating agents; anti-CD31 aptamer (APT-coated) and anti-CD31 antibody (Ab-coated). Three constructs were fabricated for each group and the scaffolds were cultured within a bioreactor for 7 days. Scale bar, 200 μm. (B) Schematic diagram of the strategy illustrating the recellularization of the decellularized rat liver constructs with HepG2 cells, LX2 cells, HUVECs and MSCs. Representative immunohistochemical images of CTL-VBHL, APT-VBHL and Ab-VBHL constructs stained with α-SMA (red). CFDA-labeled HUVECs (green) and nuclei (blue) were also detected. Scale bar, 100 μm. (C) Schematic diagram of the strategy used to implant VBHL constructs into the TAA-induced cirrhotic rats. After 8 weeks of TAA induction, the rats received heterotopic implantation of decellularized liver matrix (DLM implanted) or VBHL constructs (CTL-VBHL; CTL implanted, APT-VBHL; APT implanted, Ab-VBHL; Ab implanted). (D) Representative H&E and picrosirius red staining images of harvested host liver tissue sections after 4 weeks of implantation. Scale bar, 40 μm. Adapted from the study by Kim et al. with permission of Biomaterials, copyright 2023 [116].

Vascularized bioengineered human livers (VBHL) were also fabricated using decellularized liver [116]. Decellularized liver constructs were coated with anti-CD31 aptamer (APT-coated) or anti-CD31 antibodies (Ab-coated), seeded with HUVEC cells and subjected to bioreactor culture for 7 days. The HUVECs in the presence of the anti-CD31 aptamer coating formed continuous endothelium along the vascular lumens, promoting more efficient endothelization through the liver construct than the anti-CD31 antibody (Fig. 3A). To repopulate the liver scaffolds with the parenchymal and non-parenchymal cells, different delivery routes and seedings were employed at different times by cannulating both the bile duct and portal vein. After additional 7 days of culture, the αSMA-positive cells, regarded as integrated MSCs, were distributed around the perivascular region of the anti-CD31 aptamer-coated vessels and interconnected with ECs, unlike the anti-CD31 antibody coated vessel which was unable to form complete vascular-like structures (Fig. 3B). *In vivo* evaluation of the VBHL scaffolds was also investigated in a rat hepatic cirrhosis model induced by TAA administration and implantation into the interlobular space of the liver (Fig. 3C). H&E and picrosirius red staining showed that the APT-VBHL constructs attenuated hepatic fibrosis compared to the decellularized liver matrix (DLM) and sham groups which showed eosinophilic changes and interlobular septal thickening (Fig. 3D). The study showed that reconstruction of a vascularized liver construct using anti-CD31 aptamer coating that promotes the re-endothelization supported liver functions in a rat model of liver fibrosis.

### 5.3 Lung

Lung decellularization routes include both the airways and vasculature and are focused on the restoration of proper gas exchange for function and regeneration. Several groups have explored lung decellularization by means of perfusion of various decellularization agents including SDS, Triton X-100, and CHAPs [[117], [118], [119], [120]]. In 2010, Ott et al., performed pulmonary arterial perfusion of 0.1% SDS and 1% Triton X-100 over the span of 72 h [121]. Whole lungs repopulated with HUVECs and rat fetal lung cells demonstrated repopulation of entire pulmonary scaffolds; however, ultimately presented with pulmonary edema when tested *in vivo*. Since then, many of the early studies demonstrated ability to support elementary organ function, but caused microstructural damage to both the airways and vascular network leading to pulmonary leakage and limited function [122,123].

One recent study by Young et al. investigated the need for dlECM enhancement with basement membrane proteins following decellularization for proper epithelial and alveolar barrier formation [124]. *In vitro* coating of dlECM supplemented with laminin or fibronectin demonstrated superior alveolar epithelium barrier function. This increased barrier resistance was associated with an up-regulation of junction proteins, including Claudin-18, which may play a role in the stabilization of the alveolar barrier. In another study, Obata et al. investigated the use of a natural fatty acid, soap potassium laurate (PL) as an alternative to the harsh detergent SDS [125]. Rat lung decellularized with PL demonstrated cellular removal with improved preservation of architecture (elastin microfibrils, sGAG and ECM proteins) compared to SDS decellularization. PL-decellularized scaffolds also showed increased uniform distribution of rat epithelial cells. Furthermore, few recent studies also demonstrate the effect of Epac agonist in improved endothelial barrier function with increased junction proteins, which can be useful for improved vascular barrier formation [124,126].

Further research is needed to advance decellularization and recellularization techniques to minimize lung ECM component loss and ECM damage. This is to allow for appropriate cell distribution upon recellularization of the airways and vasculature for gas exchange, compliance, and proper *in vivo* lung function.

### 5.4 Kidney

Like whole lung decellularization, kidney decellularization is commonly achieved via the vasculature, namely the renal artery as well as the ureter. In 2009, Ross et al. reported the first decellularization of whole rat kidneys in which decellularization was achieved through arterial perfusion of various agents including Triton X-100, SDS, sodium deoxycholate and DNase [127]. Post-recellularization with murine embryonic cells, cells exhibited apoptosis forming lumens and progressive loss of embryonic features suggesting differentiation. Recently, research has been focused on minimizing damage to the renal microstructure and vascular for improved re-endothelization and creatinine/urine production for functional kidney development [[128], [129], [130], [131]].

For maintenance of vascular integrity of acellular kidney scaffolds, two different treatment protocols (SDS + DNase or Triton X-100 + SDS) were employed for the decellularization of pig kidneys for a total of 97 or 144 h, respectively [132]. Triton X-100 + SDS treated kidneys demonstrated improved intact microvascular architectures with vascular patency in comparison to the other protocol which presented disrupted vascular morphology and led to blood extravasation. However, for re-endothelization with MS1 endothelial cells, both protocols showed decreased platelet adhesion resulting in blood vessel thrombosis. In a recent study, decellularization of sheep kidneys was performed by perfusion of Triton X-100 in combination with SDS or SDS only [133]. Treatment of SDS only led to extravasation and blood leakage *in vitro* and *in vivo* in a sheep model due to poor vascular integrity that was otherwise not detected in the other treatment and demonstrated vascular integrity and function for up to 12 h. In another study, rat kidney grafts were decellularized by renal artery perfusion of SDS for 6 h. Decellularized grafts exhibited intact scaffold microarchitecture of the glomeruli and tubules with an intact blood vessel integrity [134,135]. When infused with human induced pluripotent stem cell-derived endothelial cells, histological examination demonstrated recellularization within the cortical region of the kidney, distributed in the arterial structure and glomerular capillaries with cell maintained expression of endothelial marker CD144 [135].

**Fig. 4:**
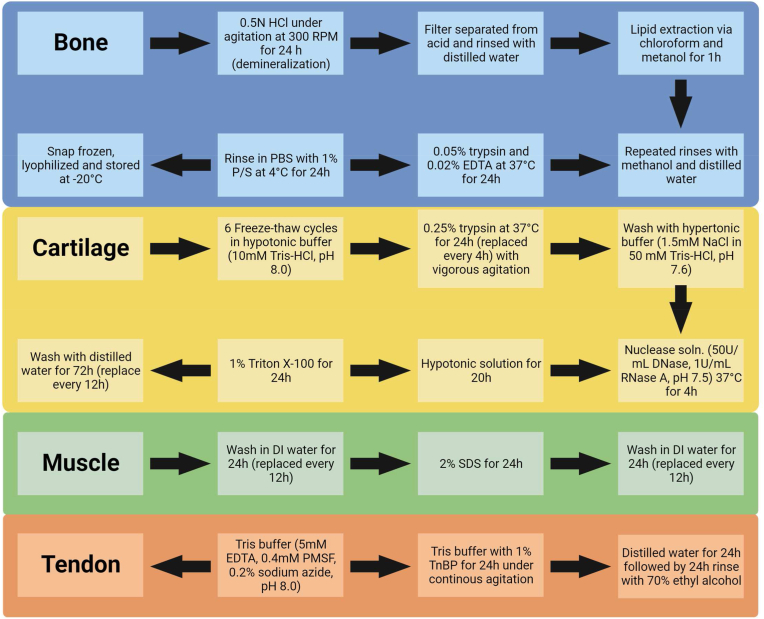
Musculoskeletal tissue decellularization protocols. Commonly employed decellularization techniques/protocols for bone, cartilage, muscle, and tendon tissues. These protocols utilize the combination of physical, chemical and enzymatic decellularization methods to achieve better outcomes in terms of DNA removal while preserving tissue mechanical properties and bio-chemical composition.

Although most initial and current studies focused on perfusion-based treatment for whole organ decellularization, other tissues do not contain a vascular network to allow for these methods, therefore other decellularization approaches and methods were developed. The most common methods developed utilized agitation, but immersion, pressure gradient systems, and supercritical fluids were employed as well [136]. Using these methods, decellularization of different tissues was employed including cartilage, bone, muscle and tendon. Commonly employed decellularization techniques (combination of physical, chemical and enzymatic) for bone, cartilage, muscle and tendon tissues are shown in Fig. 4.

### 5.5 Bone

As an alternative to bone grafting, decellularized bone derived scaffolds have been investigated for bone repair strategies. Early studies focused on the demineralization of bone using an acidic treatment for the removal of mineral components whilst leaving proteins, calcium-based solids, inorganic phosphates and some trace cell debris [137,138]. Later studies also focused on the removal of cellular constituents from the source, known as decellularization [139,140]. Since then researchers have investigated the osteoconductive potential of decellularized bone ECM (dbECM) to be used as a natural bioactive biomaterial [141]. Numerous studies have demonstrated the osteoinductive ability of the dECM matrices which can induce osteogenic differentiation and bone formation *in vitro* and *in vivo* [[142], [143], [144]].

Additionally, decellularized bECM is often used in combination with collagen, hydroxyapatite (HA), BMP and other relevant GFs for enhanced osteogenesis and bone formation [[145], [146], [147]] [[145], [146], [147]]. In one study, decellularized bone scaffolds were coated with a collagen/HA mixture and loaded with SDF-1α [148]. The results of the study demonstrated enhanced osteogenesis of MSCs *in vitro* and evidence of recruitment of endogenous stem cells when subcutaneously implanted. In another study, Rindon et al. developed heparin conjugated dbECM particles tethered with PDGF-BB (HP-DCB-PDGF) in order to promote sustained release of the growth factor and provide synergistic osteogenic cues [149]. Compared to grafts without PDGF-BB, all grafts with the growth factor exhibited increased osteogenic differentiation with HP-DCB-PDGF presenting significantly greater calcium deposition compared to grafts containing only PDGF by ASCs *in vitro*.

Furthermore, hydrogels fabricated from decellularized bones have also been investigated through solubilization of dbECM [140,143,150]. Alom et al. studied the osteogenic potential of dbECM in the presence and absence of osteogenic medium [150]. Immunocytochemistry staining revealed higher levels of osteogenesis specific proteins, OPN and OCN expressed by mouse primary calvaria cells when cultured on dbECM hydrogels in both osteogenic and basal medium. However, when cultured on collagen type I hydrogels or TCP, cells only expressed OPN and OCN when cultured in osteogenic medium, indicating the osteogenic potential of dbECM without osteogenic supplements. Similar results were also demonstrated by Paduano et al., in which cells cultured on dbECM hydrogels had a significant up-regulation of RUNX-2 and BSP in the absence of osteogenic inducers compared to when cultured on Col-I hydrogels [143]. The bone regeneration capacity of non-tissue specific ECM was also evaluated. In this study, injectable hydrogels were developed from decellularized porcine skin incorporated with biphasic calcium phosphate powder (BCP) [151]. Biochemical analysis of these hydrogels revealed retention of collagen and GAG content after solubilization along with trace amounts of VEGF and BMP-2. *In vivo* evaluation demonstrated increased bone formation at 8 weeks in hydrogels containing BCP (ECM-BCP) compared to those that did not. Moreover, the hydrogels showed evidence of bone formation by endochondral ossification by bridging and connecting the fracture ends with collagen cluster and bone formation colocalized with osteoblasts/osteoclasts after 4 and 8 weeks, respectively. As hydrogel use in bone tissue repair is often limited due to their low mechanical properties, hydrogel reinforcements must be considered for their use in load-bearing settings.

### 5.6 Cartilage

In 2010, Yang et al. describe the decellularization of cartilage powder by chronological treatment with trypsin, nucleases, hypotonic buffer and Triton X-100 [152]. The acellular powder was then crosslinked with UV irritation and when seeded with MSCs showed good biocompatibility. Evaluation of the tissue demonstrated cell fragment and DNA removal, however, witnessed some level of disruption in terms of cartilaginous structure and mechanics. Since then efforts have been focused on improvements in decellularization methods/protocols for limited disruption to the native architecture/loss of cartilaginous ECM proteins for improved chondrogenesis and mechanical function [[153], [154], [155]] [[153], [154], [155]] [[153], [154], [155]].

Recent research has focused on improvements in the treatment methods needed to successfully decellularize the intrinsic dense and compact structure of cartilage. In one study, cartilage sheet samples were decellularized using two different treatments of SDS or Triton X-100 [25]. By preparing thin sheets, the tissue was decellularized using gentle treatment compared to traditional treatments and even displayed presence of growth factors including TGF-β1, IGF-1 and BMP-2. However, depending on the application, these sheets may not contain adequate mechanical properties. The application of ultrasonic bath was also explored to improve the penetration of decellularization reagents, however requires further advancements to improve the decellularization efficacy [156].

Various studies focused on scaffold structure development and approaches for mechanical enhancement of dcECM including different crosslinking methods, composite scaffolds, or electrospun and thermoplastic reinforced scaffolds [[157], [158], [159]] [[157], [158], [159]] [[157], [158], [159]]. Browe et al. developed decellularized cartilage ECM-derived (dcECM) scaffolds that were crosslinked using glyoxal and dehydrothermal treatment [160]. These scaffolds supported cartilage ECM synthesis when seeded with fat pad derived stromal cells (FPSCs) and displayed high elastic properties when evaluated by compression tests. However, the mechanical properties were still inadequate compared to native AC and noted the need for additional mechanical support. One strategy developed electrospun gelatin-polycaprolactone nanofibers and dcECM composite scaffolds and showed that the integration of the nanofibers significantly increased the mechanical properties while the dcECM led to increased secretion of cartilage-specific proteins [161]. While another study fabricated scaffolds by combining PLGA and decellularized cartilage powder via solvent casting/salt-leaching technique followed by EDC/NHS mediated crosslinking [162]. Scaffolds produced by this method revealed increased compressive strength (0.89 MPa), which is comparable to native cartilage.

**Fig. 5:**
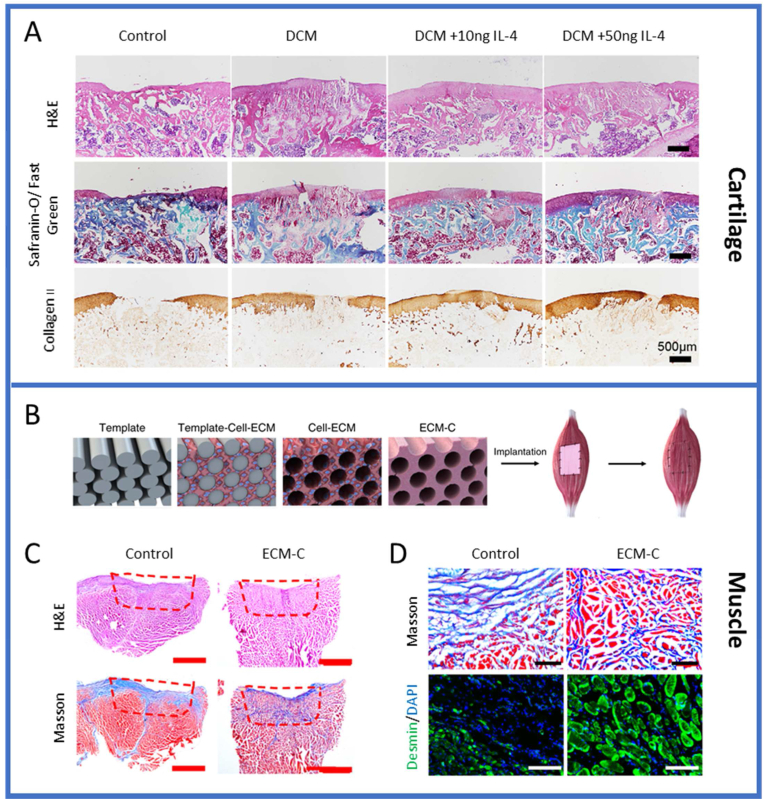
*In vivo* evaluation of the decellularized tissue biomaterials/grafts for tissue repair and regeneration. (A) Histological evaluation of *in vivo* cartilage regeneration after 8 weeks. Safranin O- Fast green and immunohistochemical staining of COL II of repaired cartilage in different groups after 8 weeks. Adapted from the study by Tian et al. with permission of Acta Biomaterialia, copyright 2023 [165]. Muscle regeneration of the rat tibialis anterior (TA) muscle defects treated with ECM-C or control scaffolds, (B) Fabrication of ECM-C and control scaffolds. The schematic diagram of the PCL fiber template, template-cell-ECM, cell-ECM, ECM-C and control group during the preparation process and implantation. (C) Macroscopic view of regenerated TA muscle defects by staining the cross-sections with Masson trichrome and H&E. (D) Microscopic images of the cross-sections of regenerated TA muscle at 1 month, stained with Masson trichrome and immunofluorescently stained for desmin. Adapted from the study by Zhu et al., open access, https://creativecommons.org/licenses/ [166].

Meanwhile there are a few attempts to process cartilage as a whole tissue. Luo et al. investigated the decellularization of whole cartilage explants by introduction of channels to allow for infiltration of decellularization agents and subsequent recellularization [154]. Results showed ∼90% DNA reduction with near complete sGAG removal, little increase in porosity of the tissue and decreased mechanical properties. When reseeded with FPSCs, the channels supported cell viability however, demonstrated limited cell migration into the explant ECM and thus inadequate recellularization. In efforts to improve this, another study utilized laser surface engineering for creation of micropores onto the surface of cartilage implants [163]. After 8 weeks of culture, these laser-modified scaffolds exhibited improved cell attachment and evidence of ECM deposition on the surface and within the micropores by rabbit chondrocytes *in vitro,* which is attributed to enhanced porosity and surface roughness. Recently, Golebiowska et al., developed a rapid protocol to decellularize articular cartilage while retaining ECM and biochemical components including GAGs, Collagen II and some of the growth factors native to articular cartilage [164]. Decellularized cartilage matrix (DCM) was developed and combined with intra-articular injection of M2-polarizing cytokine IL-4 and assessed in an *in vivo* osteochondral rat defect model [165]. Evaluation of the osteochondral defect regeneration showed that compared with the control group (no implant), partial hyaline cartilage regeneration was achieved in the DCM group after 8 weeks, and a specific dose of IL-4 (10 ng) achieved a better repair effect (Fig. 5A). The Safranin O and fast green staining showed that the DCM +10 ng IL-4 group promoted regeneration of hyaline-like tissue at 8 weeks and the effect of subchondral bone reconstruction was better than that of the other groups. Immunohistochemical results also showed that collagen type II deposition increased in all the DCM groups, with DCM+ 10 ng IL-4 outperforming the other groups. The study demonstrated that the immunomodulatory effects of the cell-free DCM scaffold using IL-4 could achieve cartilage regeneration in a rat knee osteochondral defect model.

### 5.7 Muscle

Decellularized ECM has also been applied to skeletal muscle, specifically in cases of irreversible volumetric muscle loss (VML). Many current studies have made developments in micro-/nano-architecture fabricated constructs to mimic the native myofiber alignment and network structure [167,168]. In one study, Choi et al. developed sinusoidal wavy polystyrene surfaces (wavelengths 20, 40 and 80 μm) coated with decellularized muscle ECM [169]. The combination of the two led to the formation of multinucleated myotubes that were well aligned as well as exhibited enhanced myogenic differentiation compared to collagen-coated and non-coated substrates. In another study, electrospun scaffolds were developed of aligned nanofibers of PCL and decellularized muscle tissue [170]. Unidirectional alignment of nanofibers supported primary satellite cell proliferation and differentiation *in vitro*. When tested in a murine model, increased myofiber regeneration was observed at day 7 and 28, however, with limited improvements in muscle force production, possibly owning to the need of longer time-points for evaluation [171]. Results from these studies indicate the influence of topographical and biochemical cues on cellular behavior and promoting myogenic activity.

Further studies have utilized exogenous GFs to enhance cellular recruitment and infiltration and aid in functional muscle regeneration. In one study, macroporous sponges were developed from decellularized mECM with chemically immobilized SDF-1α within the constructs [172]. These constructs showed significantly higher infiltration of muscle-derived stem cells with better distribution compared to no SDF-1α. *In vivo* results showed increased infiltration of CXCR4 + cells as well as significantly increased number of vessels, indicating higher induction of angiogenesis. In another study, a biofunctionalized scaffolding system was developed consisting of decellularized mECM and IGF-1 [173]. *In vitro* testing showed that these scaffolds led to higher cellular infiltration of C2C12 cells as well as higher myosin heavy chain expression and myotube formation compared to the control groups [173,174]. Moreover, these cell-free scaffolding systems were also tested in rabbit tibial anterior muscle defect models and demonstrated higher host cell infiltration and greater number of muscle fiber formation compared to collagen and dECM groups [174].

In order to improve the homogenous decellularization and preservation of architecture and bioactive cues/functional capacity, perfusion-based methods have also been adopted for large and thick tissues, such as muscle tissue. Through perfusion of enzymatic and detergent treatments, Zhang et al. decellularized porcine rectus abdominal muscles [175]. The obtained tissue retained the intricate architecture and internal vasculature while also preserving bioactive components and mechanical properties compared to native tissue. ECM scaffolds with aligned microchannels were also developed and assessed *in vivo* for skeletal muscle defect regeneration [166]. ECM scaffolds were engineered with parallel microchannels (ECM-C) by subcutaneous implantation of sacrificial templates, followed by template removal and decellularization (Fig. 5B). Histological staining showed a large number of cells infiltrated the interior of the scaffold within the ECM-C group with the deposition of large amount of new ECM (Fig. 5C). Selected regions stained either with Masson trichrome or for desmin revealed the presence of a high density of neo-muscle fibers within ECM-C, while only sparse neo-muscle fibers were seen around the control scaffolds-treated defects (Fig. 5D). These studies demonstrate the potential use of decellularized ECM-based scaffolds for various tissue repair and regeneration strategies.

### 5.8 Tendon

The burdens of tendon injuries have also caused researchers to create bio-functional and bio-mechanical tendons for injury repair to which decellularized grafts represent promising alternatives [176]. Due to the dense compact structure of tendons, thin sheets or slices have largely been used and developed for enhanced decellularization and subsequent recellularization/infiltration efficacy [177,178]. In one study, book-shaped scaffolds were prepared by stacking multi-layers of decellularized tendon slices and BMSC sheets [179]. *In vitro* studies revealed homogenous distribution and alignment of cells as well as an up-regulation of tendon-related genes, including tenomodulin and Alpha-1 collagen type I, compared to the control. In another study, multilayer decellularized tendon slices were prepared for reconstructing large rotator cuffs in rabbit models [180]. Results showed that these grafts promoted host cell ingrowth along with fibrocartilage and bone formation at the tendon-bone interface that led to improvements in the mechanical properties.

In order to improve homogenous cellular distribution (whilst reseeding or cellular infiltration), dynamic culture/mechanical stimulation has been explored recently [181,182]. For the purpose of replicating mechanical properties of the native ACL, Lee et al. subjected tendons to simultaneous tension and torsion in a bioreactor through biaxial cyclic loading [183]. The use of the bioreactor led to significantly increased expression of tendon-specific genes and ultimate tensile load and stiffness of the recellularized tendons. In a recent study, tendons were seeded with MSCs and subjected to bidirectional perfusion and stretch cycling [184]. The use of this bioreactor demonstrated similar results with homogenous distribution of cells with superior production and organization of newly formed collagen compared to static culturing.

Similar to decellularized muscle applications, decellularized tendon scaffolds have also been incorporated with exogenous GFs for enhanced tenogensis including TGF-β3 and BMP-12 [185,186]. Few decellularized triphasic scaffolds have also been developed for tendon to bone ruptures for enthesis repair [187,188]. In these studies, tri-phasic tendon-fibrocartilage-bone interfacial tissue constucts were developed to facilitate tendon-to-bone healing. In a recent study, a gradient book-type triphasic scaffold was developed and showed superior osteogenic, chondrogenic and tenogenic inducibility in the respective regions [189]. When implanted in a rabbit BTI injury model, the scaffolds showed accelerated healing with attained triple biomimetic structure and cellular distribution.

## 6 Improved decellularization methods

**Fig. 6:**
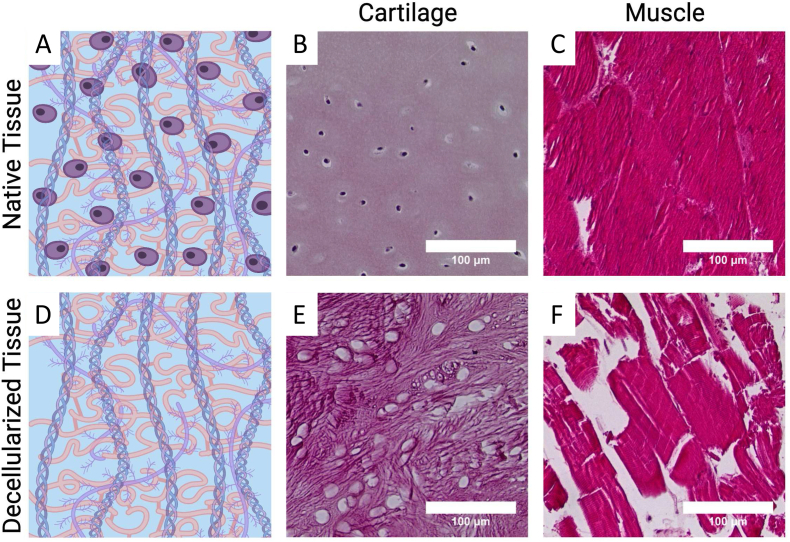
Schematic of native tissue containing various ECM components and cells (A) and decellularized tissue void of cells (D). Histological H&E stained images of native cartilage tissue (B), native muscle tissue (C), decellularized cartilage tissue (E) and decellularized muscle tissue (F). Native tissue demonstrating intact extracellular matrix with presence of cells while decellularized tissue demonstrating lack of cell nuclei presence with some disruption in the matrix structure. Part of this figure is adapted from the study by Golebiowska et al. with permission of Annals of Biomedical Engineering, copyright 2023 [164].

Due to the large variety of tissues/organ sources and protocols investigated thus far, standard criteria for establishing decellularized tissues have been proposed [92,190,191]. In general, these include the absence of visible cell nuclei and removal of DNA content which is critical to minimize the potential adverse host responses to ECM products [192]. Along with assessing the removal of cellular and genetic material, the perseveration of proteins and other ECM components such as collagen, glycosaminoglycans and GFs as well as mechanical properties is also required (Fig. 6) [190]. Maintaining the native milieu of biochemical and biomechanical cues is essential for providing proper signaling that are required for governing cell behavior and cell-cell/cell-ECM interactions for recellularization and restoration of functional tissues/organs.

Long exposure times to harsh decellularization reagents cause significant reduction in ECM components [193,194]. Recent efforts have been in the area of improving decellularization outcomes via use of milder detergents and other reagents (e.g. salt solutions and enzymes), pressure-/agitation-assisted and advanced decellularization techniques [195]. Mazza et al., first described decellularization of liver left lobe in 2015 [196]. Decellularization was a perfusion-based regime consisting of different reagent treatments including: Trypsin-EDTA, SDS, Triton X-100, peracetic acid and ethanol. Decellularized liver scaffolds showed significant DNA reduction and no evidence of cellular debris as well as architecturally preserved tissue. This protocol however took 2–6 weeks to complete and thus is not ideal for clinical applications. Efforts by Mazza et al. continued again in which they describe rapid decellularization of liver cubes (125 mm^3^), conducted by employing different agitation speeds (g-force intensities) [197]. At high g-force values (45g), liver tissue cubes turned translucent in 3 h with removal of cellular material and preservation of ECM components confirmed by H&E staining and SR and Elastin Van Gieson staining, respectively. These studies demonstrate rapid protocol employment through utilization of high shear stress for liver ECM preservation and minimization of detergent/reagent exposure and processing times.

Similarly, Golebiowska et al., developed a rapid decellularization for articular cartilage tissue decellularization (cite). Decellularization of cartilage tissue was performed through a series of physical, chemical and enzymatic treatments. Modifications in the exposure to harsh treatments, specifically trypsin and Triton X-100, led to the rapid decellularization of cartilage tissue to nearly ¼ of the total time to implement. H&E staining and dsDNA quantification confirmed the removal of cellular components. Additionally, the modifications to the protocol led to a higher retention of biochemical and ECM components confirmed by sGAG and hydroxyproline quantification. The authors speculate that the rapid protocol developed by reducing the exposure to enzymatic and chemical treatment may aid in the retention of GFs and other signaling molecules important for repair and regeneration. These can serve as guiding protocols for future work where existing decellularization protocols can be revisited with the goal of preserving native structural and biochemical components.

Numerous decellularization procedures have been investigated, however, there is currently no consensus regarding optimal methods, as this is often tissue-/source- and application specific. While many decellularization procedures often use harsh treatment methods that result in the successful removal of cellular remnants, they also lead to varying effects on the dECM constructs. These include damage to/reduction of structural and signaling proteins, significantly compromising the integrity and performance of dECM constructs [198]. Given that native tissues contain the necessary tissue-specific factors, proteins, and 3D ultrastructure for residing cells, there is a need for improved and optimized decellularization techniques that minimizes the impairment of the ECM tissue and components during processing.

As improvements in decellularization processing techniques continues, some of the fundamental/basic questions are still unclear. Decellularized ECM products have varying relevant physical and chemical cues post-processing, with no criteria present based on the retention of those cues/properties that might led to better regeneration. These products are often repopulated with cells or rely on cellular infiltration, acting not only as a cell carrier but also as a complex biologically relevant scaffold for regulating cellular behavior and tissue regeneration [199]. Still, there is limited knowledge on the specific components and their concentration that is associated with and required for tissue regeneration.

## 7 Decellularized ECM-induced chemotaxis

An *in situ* regeneration approach that utilizes the body's own regenerative capacity by mobilizing host endogenous stem cells or tissue-specific progenitor cells to recellularize grafts is a promising cell-free approach for TE. The mobilization of endogenous cells relied on in these approaches is through a process known as chemotaxis. Chemotaxis is the directional migration of cells in response to a gradient of soluble chemoattractants [200]. The migration is induced by homing signals that are released and is driven by a number of growth factors and chemokines. TE approaches that rely on cellular recruitment or chemotaxis can allow for the development of cell-free strategies and overcome the limitations of using exogenous cells including reduced FDA approval difficulties/hurdles associated.

Decellularized ECM has the ability to retain native counterpart constituents, including structural components and biochemical signaling molecules such as growth factors [201,202]. Demineralized bone matrix has long been used as bone graft substitutes due to their osteoinductivity that has been attributed to the presence of growth factors that are detected including BMP, IGF-1 and TGF-β [203]. More recently studies have investigated the instructive ECM elements that are preserved in dECM. Detergent decellularized human kidney demonstrated the presence of a number of heparin-binding growth factors, including FGF2, VEGF, BMP-2, HGF, EGF, PDGF-bb and TGF-β, although these were at reduced levels compared to native tissue [201,204,205]. Other studies have demonstrated similar results with the preservation of GFs in decellularized porcine decellularized mesothelium including VEGF, FGF and TGF-β, which stimulated human fibroblasts to produce more VEGF compared to fibroblasts grown on TCP [206].

**Fig. 7:**
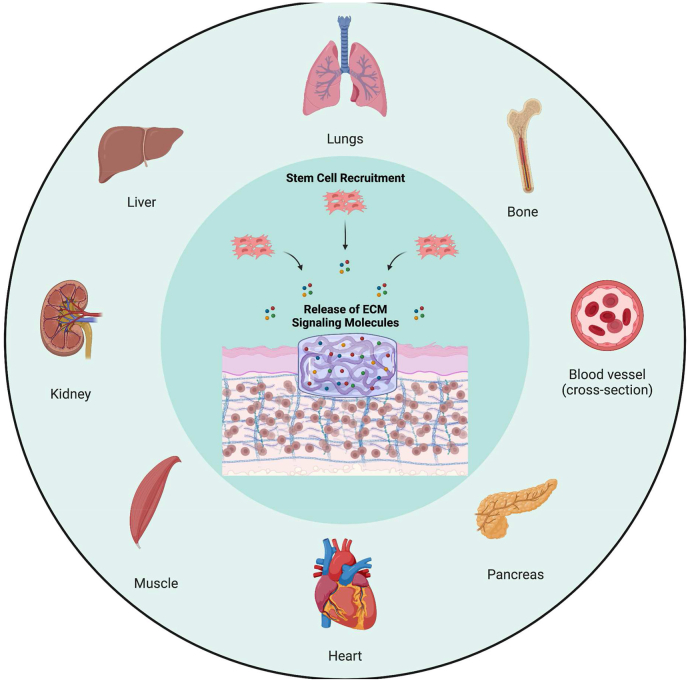
Schematic demonstrating chemotactic ability of the decellularized tissue or cell-derived extracellular matrix. Directed or oriented movement of cells in response to a chemical stimulus or chemoattractant is referred to as chemotaxis. Utilization of dECM biomaterial or grafts can provide native tissue-specific bioactivity and offer biomimetic framework consisting of the major ECM components to mimic an *in vivo* microenvironment as well as can contribute to endogenous recruitment through their release of bioactive cues. Such strategies can stimulate an *in situ* tissue regeneration response as a cell-/growth factor-free tissue engineering strategy for the repair and regeneration of various tissues. The inner circle shows cellular recruitment by the implanted decellularized cartilage tissue, while the outer circle is depicting various tissues and organs for which the same concept can be applied to achieve cellular migration (or chemotaxis) needed for repair/regeneration.

The bio-instructive signaling cues present in the dECM-based grafts may provide tissue-specific cues for directing cellular behavior and orchestrating cellular chemotaxis (Fig. 7) [207]. Towards these efforts, reinforced composite scaffolds consisting of decellularized ECM microparticles in a hyaluronic acid-based hydrogel were developed for cellular signaling [208]. Results showed that within 48 h, primary chondrocytes receullarized the particles and maintained chondrogenic phenotype via gene expression analysis. Others have developed cell-derived matrices (CDMs) in the form of cell sheets decellularized with different concentrations of SDS. Treatment with lowest SDS (0.5%) led to preserved bioactive components of the cell-laid ECM, increased the recruitment of MSCs and improved regeneration of osteochondral defects in rabbits [209]. Additionally, zebrafish cardiac ECM was decellularized, lyophilized and resuspended in normal saline [210]. When used for cell migration studies, the dECM demonstrated prominent migration of human cardiac stem cells and human heart perivascular MSCs when cultured under nutrient-deprived culture conditions (25% complete media and 2.5% FBS). These studies suggest that the preservation of ECM exhibits bioactivity post-decellularization and chemotactic effects; however, limited studies are available regarding chemotactic ability of decellularized ECM [211].

**Fig. 8:**
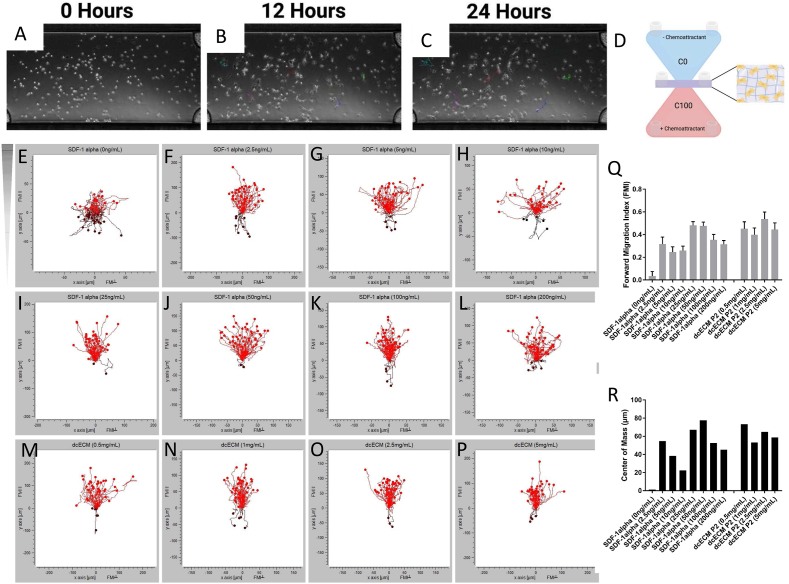
MSC Chemotaxis induced by the decellularized cartilage gel. Representative time course images of collagen gel-embedded cells in response to decellularized cartilage ECM immediately after seeding (A), at 12 h (B) and 24 h (C) with overlaid cell migration trajectories. (D) 3D chemotaxis assays with live cell imaging. Migration tracks of human MSCs in response to (E–L) SDF-1alpha, (M–P) dcECM P2. Forward migration index (FMI) parallel to the gradient of hMSCs migrating in response to SDF-1alpha and dcECM (Q) (n = 40) and the center of mass of cellular endpoints from trajectory plots (R). Adapted from the study by Golebiowska et al. with permission of Annals of Biomedical Engineering, copyright 2023 [164].

Our own laboratory utilized 2D and 3D chemotaxis assays and live cell imaging to track the cellular migration in response to decellularized cartilage ECM. Articular cartilage was decellularized, solubilized and the chemotactic activity of the tissue-derived gel was investigated [164]. The results showed that the tissue-derived dECM retained biochemical cues of the native tissue. Moreover, the results demonstrated the ability of the decellularized cartilage ECM to stimulate migration of hBMSCs in both 2D and 3D model systems which was at similar levels compared to a known MSC chemoattractant (Fig. 8). Furthermore, the antagonist studies also demonstrated that the directed migration of the hBMSCs was likely attributed to the GFs presence/SDF-1α induced.

These studies demonstrate the ability of decellularized tissues to induce cellular migration and affect cellular behavior. These grafts have the potential to be used as a strategy for guided tissue regeneration (GTR) by providing bioactive cues within the scaffold to induce or allow for cellular recruitment to drive tissue regeneration. These regenerative approaches allow for the design of ECM-based grafts to trigger chemotaxis without the need for cellular transplantation to drive regeneration. However, further studies are needed to investigate the retained growth factor types and amounts and their bioactivity assessment. With little or no externally added growth factors or signaling molecules, decellularized tissue biomaterials can be clinically used for *in situ* tissue engineering, where the repair and regeneration is guided by the dECM biomaterials/grafts. This can lead to a new paradigm in tissue engineering.

## 8 Decellularized tissue/graft mechanical performance

In addition to improved retention of compositional components of decellularized ECM based biomaterials, the mechanical properties are critical. Differences/mismatches in mechanical properties between TE grafts and native tissue can lead to stress concentrations at the implant-tissue interface and mechanical failure. Maintaining the mechanical integrity of the ECM is important to ensure its proper functionality, often evaluated as elastic modulus, ultimate tensile/compressive strength, and yield strength. These properties are largely provided by the structural ECM proteins such as collagen, elastin, fibronectin and laminin [212].

The decellularization process has an impact on these proteins/ECM structures, affecting the mechanical properties of these materials and thus can have negative impacts/limited success. Minimizing the damage to the microstructure and mechanical integrity of the remaining dECM products is therefore necessary for structural applications. Table 3 summarizes the effect of decellularization on mechanical properties. For example, Partington et al. decellularized tracheas by multiple cycles of detergent-enzyme treatments. The resulting decellularized tissues had reduced collagen and GAG content levels and mechanical testing evaluation showed decreased tensile strength [213]. The retention levels of these proteins must be optimized based upon the tissue mechanics necessary for function.

**Table 3:** Decellularized tissue/graft mechanical properties in comparison with the native tissue.

Tissue	Mechanical Testing	Property Assessed	Native Tissue	dECM	Ref
*Trachea*					
	Tensile Testing	Elastic Modulus	2.68 MPa	2 MPa	[283]
*Cartilage*					
	Compression Testing	Instantaneous Compressive Modulus	4 MPa	0.6 MPa	[284]
	Compressive Strength Testing	Elastic Modulus	0.36 ± 0.07 MPa	0.55 ± 0.15 MPa	[285]
	Tension-Torsion Compression Testing	Ultimate Tensile Strength	2.10 ± 0.29 MPa	1.90 ± 0.31 MPa	[286]
		Elastic Modulus	18.40 ± 4.02 MPa	16.49 ± 4.79 MPa	
	Stress-Relaxation Testing	Equilibrium Modulus	∼148 kPa	∼20 kPa	[154]
*Bone*					
	Unconfined Compression Testing	Elastic Modulus	198.2 ± 159.5 MPa	144.9 ± 101.3 MPa	[287]
	Compression Testing Three-Point Bending Testing	Elastic Modulus	Radius: 7 ± 1 GPa Ulna: 6 ± 1 GPa	Radius: 7 ± 3 GPa Ulna: 9 ± 3 GPa	[288]
		Compressive Strength	Radius: 173 ± 4 MPa Ulna: 125 ± 24 MPa	Radius: 116 ± 42 MPa Ulna: 129 ± 39 MPa	
		Yield Strength	Radius: 168 ± 5 MPa Ulna: 120 ± 26 MPa	Radius: 113 ± 41 MPa Ulna: 124 ± 40 MPa	
Bone-Fibrocartilage-Tendon (BFT)	Uniaxial Tensile Testing	Elastic Modulus	501.48 ± 91.56 MPa	384.97 ± 86.37 MPa	[188]
		Ultimate Tensile Strength	53.27 ± 11.84 MPa	45.41 ± 7.05 MPa	
		Failure Strain at UTS	0.143 ± 0.009	0.129 ± 0.014	
*Muscle*					
	Stress-Relaxation Testing	Tangent Modulus	308 ± 51.1 kPa	218.24.5 kPa	[289]
	Uniaxial Tensile Testing	Elastic Modulus	0.19 ± 0.07 MPa	0.41 ± 0.32 MPa	[290]
		Ultimate Tensile Strength	0.21 ± 0.08 MPa	1.32 ± 0.85 MPa	
		Strain at Failure	1.24 ± 0.27	160 ± 0.30	
	Uniaxial Tensile Testing	Elastic Modulus	2.04 ± 0.54 MPa	1.74 ± 0.46 MPa	[291]
		Ultimate Tensile Strength	0.19 ± 0.03 MPa	0.23 ± 0.04 MPa	
		Strain at UTS	1.23 ± 0.44	1.38 ± 0.27	
*Tendon*					
	Tensile Testing	Elastic Modulus	299.71 ± 41.67 MPa	210.68 ± 46.43 MPa	[292]
		Ultimate Tensile Strength	34.67 ± 3.47 MPa	29.69 ± 6.73 MPa	
		Strain at UTS	15.50 ± 3.12%	19.16 ± 4.58%	
		Stiffness	25.50 ± 3.71 N/mm	27.40 ± 8.66 N/mm	
	Ultimate Tensile Stress Testing	Yield Strain	8.337 ± 0.142%	7.829 ± 0.442%	[293]
		Failure Strain	8.712 ± 0.510%	8.851 ± 0.216%	
		Yield Stress	5.774 ± 0.44 MPa	5.782 ± 0.775 MPa	
		Failure Stress	6.029 ± 0.414 MPa	6.045 ± 0.759 MPa	
		Elastic Modulus	76.13 ± 4.12 MPa	70.31 ± 5.91 MPa	
	Ultimate Load to Failure Testing	Ultimate Load	200.39 ± 22.11 N	185.95 ± 7.91 N	[294]
		Stiffness	44.26 ± 2.96 N/mm	45.99 ± 5.4 N/mm	
*Aorta*					
	Tensile Testing	Elastic Modulus	551.1 ± 152.2 kPa	416.5 ± 72.6 kPa	[77]

Additionally, the most challenging dECM products are hydrogels as mechanical strength is typically much lower than that of their native counterparts [140,214]. To overcome the inadequate mechanical properties of decellularized ECM-based hydrogels, many are often supplemented with synthetic biomaterials or crosslinked to tune and boost mechanical properties that were compromised during processing [87,215,216]. Understanding how processing techniques affects the tissue/graft structure physically, and how the structural changes can lead to differences in terms of mechanical properties post-decellularization must be addressed. Further efforts that preserve decellularized tissue/graft mechanical properties are warranted.

## 9 Decellularized tissue-based bio-inks (tissue inks) for 3D-printing

Biofabrication allows the ability to generate tissue analogues achieving precise 3D architectural placement for controlled pre-determined deposition of materials to attain complex geometries, pore size, etc. This technology unites cells, biomaterials, and bioactive molecules and assembles them into 3D constructs in a layer-by-layer fashion. Although numerous materials in the form of bio-inks have been developed for these purposes, many are unable to recapitulate the complexity of natural ECM for which they are designed to replace, creating less than favorable microenvironments for encapsulated cells. Thus, the use of decellularized tissue as a bio-ink or Tissue-Ink has attracted much attention in their capacity to retain biochemical cues/features of the native ECM, allowing for their ability to mimic native cell-ECM interactions through inductive cues, leading to *in vivo* tissue ingrowth for functional repair and regeneration. In other words, the use of Tissue-Ink with the above stated bioactive features can lead to Guided Tissue Regeneration (GTR), a much-desired aspect and hard to achieve with conventional biomaterials in TE.

dECM bio-inks have been shown to provide cells with the appropriate biochemical/physical cues needed to modulate cellular fate and for this reason, several types of tissue- and organ-based decellularized ECM bio-inks have been developed as tissue substitutes, including muscle, liver, heart, kidney, cartilage, tendon and skin [48,106,[217], [218], [219], [220], [221]]. The utilization of bio-fabrication technology using decellularized ECM-based bio-inks has been summarized below in Table 4. For example, Won et al. developed a printable Tissue-Ink through decellularization and subsequent pepsin digestion, solubilization and pH adjustment of porcine skin ECM [221]. Printed cell-laden constructs with human dermal fibroblasts showed 90% viability and proliferation as well as increased genes related to epidermis formation, which was attributed to the presence of bio-reactive molecules and growth factors present in the bio-ink.

**Table 4:** Biofabricated/3D printed constructs using decellularized ECM bio-inks/cell-derived matrices (CDMs).

Animal Source	Tissue/Organ	Gelation Mechanism/Crosslinking	Bio-ink Components	In vitro/*in vivo* assessment	Ref.
Porcine	Skin	Thermal gelation	Skin dECM Human dermal fibroblasts (hDF)	Supported viability and proliferation Increased epidermis formation-related genes.	[221]
Porcine	Heart	Vitamin B2-induced UVA crosslinking + thermal gelation	Heart dECM Cardiac progenitor cells (CPCs)	Supported high cell viability and proliferation with increased cardiomyogenic differentiation	[222]
Porcine	Skeletal muscles	Methacrylation	Skeletal muscle dECM C2C12 myoblasts	Supported differentiation and aligned myotube formation with increase gene expression and basement membrane component secretion	[223]
Porcine	Cartilage	Genipin	Cartilage dECM	PLGA gradient structure led to graded pore/fiber orientation structure and enhanced mechanical properties	[225]
Porcine	Tibialis anterior muscle and descending aorta	Thermal gelation	Skeletal muscle dECM + human muscle cells Vascular dECM + HUVECs	Supported high cell viability Improved vascularization, neural integration and functional recovery in VML rat model	[226]
Porcine	Hyaline Cartilage	Alginate and CaCl_2_	Cartilage dECM Human adipose-derived stem cells Alginate	Observed increased expression of chondrogenic markers. *In vivo* shape and structure maintenance with cartilaginous tissue formation after 12 weeks	[227]
Porcine	Heart	Thermal gelation	Heart dECM Primary cardiomyocytes	Enhanced cardiomyocyte maturation with aligned structural organization under dynamic culture and enhanced expression of cell adhesion molecules, integrin-based proteins, basement membrane proteins, and matrix remodeling metallopeptidases	[228]
Murine	Osteoblast/osteocyte-like cells (MLO-A5s)	–	MLO-A5 CDMs Human embryonic stem cell-derived mesenchymal progenitor cells	Improved attachment and significantly higher cell proliferation and osteogenic activity with higher angiogenic potential	[249]

Biofabricated constructs must retain its shape post-printing, degrade in a timely manner to allow for *in vivo* tissue ingrowth and contain similar mechanical properties of the surrounding native tissue. Thus, Tissue-Inks have also been modified to allow for crosslinking, which increased stability and mechanical properties. In particular, for the purpose of stabilizing and enhancing/tailoring mechanical properties of printed dECM bio-ink, authors combined vitamin B2 and UVA light and thermal crosslinking, which showed to have supported high cell viability and proliferation of cardiac progenitor cells with increased cardiomyogenic differentiation [222]. dECM bio-ink mechanical stability has also been enhanced using methacrylate photo-crosslinking process [223]. The authors combined methacrylated dECM bio-inks and PVA as a sacrificial fibrillated component to produce a uniaxially aligned micro-topographical structure with mechanically stable struts. These constructs led to the formation of a stable structure with highly aligned cell/ECM and myotube formation by C2C12 cells. Aside from the need for mechanically stable bio-inks during/immediately after the printing process, biofabricated constructs may require additional mechanical strength for certain tissues/applications, such as those requiring load-bearing properties. These mechanically enhanced constructs can be fabricated through incorporation of degradable polymers including PLA, PCL and PLGA. We used PLLA as a template structure and introduced a gel in between the polymeric filaments through selective printing, infusion, and gel printing on the template structure and its settlement into the porous structure. All three strategies supported cell placement via the gel component and their survival post-printing [9]. One strategy used PCL as a framework followed by the alternating deposition of cell-laden dECM bio-ink with varied line width of the synthetic polymer for stiffness adjustment [224]. Another used PLGA to fabricate gradient structures of low, medium and high-density grids [225]. These were thens injected with dECM + genipin crosslinker and underwent directional freezing to produce gradient oriented dECM with mechanical strength similar to that of articular cartilage.

In addition to mechanical stability, vascularization of biofabricated constructs is important for sufficient tissue ingrowth and integration with the surrounding tissue. For this purpose, Choi et al. combined muscle and vascular dECM encapsulating human skeletal muscle cells and HUVECs, respectively for the development of pre-vascularized muscle constructs for VML treatment [226]. Using co-axial printing, authors printed a compartmentalized core-shell structure, in which the vdECM served as the outer shell and the mdECM as the inner core. It was observed that these constructs led to endothelial network and myotube formation throughout the construct, which was otherwise not observed when the two cell-laden bio-inks were mixed. *In vivo* studies showed the generation of thick and densely packed newly formed muscle fibers and formation of functional blood vessels integrated with host vasculature.

Furthermore, advancements in biofabrication using tissue-specific dECM bio-ink has enabled fabrication of customizable and anatomically correct constructs for patient-specific tailored applications. Using computer-aided design and 3D fabrication, Yi et al. generated 3D models of customized nasal implants to generate the 3D exterior and interior architecture shape [227]. Afterwards, the model was injected with a bio-ink based on dcECM containing ADSCs. It was seen that tissue-derived bio-ink supported high cell viability, with higher expression of chondrogenic markers (SOX9, ACAN and COL21A) and GAG presence compared to an alginate hydrogel.

More recently, other advancements in the field include the combination of dECM biofabricated constructs with external stimulation. Heart derived ECM printed constructs underwent dynamic culture to compare the effects of culture conditioning on rat primary cardiomyocytes [228]. The hdECM and dynamic culture showed cardiomyocytes with a unidirectional, elongated and aligned structural organization with enhanced structural maturation compared to static culture or collagen encapsulated cells.

## 10 Cell-derived matrices

**Fig. 9:**
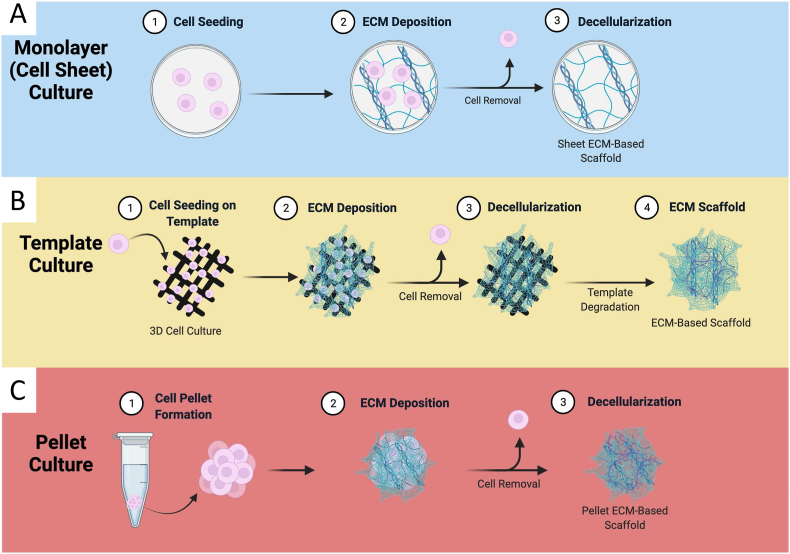
Methodologies to form cell-derived ECM. (A) Monolayer culture of cells to allow for ECM deposition, followed by decellularization to obtain cell sheets. Cell sheets can be then stacked to construct 3D tissue-like structures. (B) Cells cultured on a 3D template structure to allow for ECM deposition, followed by cell and template removal for 3D ECM scaffold. (C) Cell pellet culture and decellularization for the formation of pellet ECM scaffolds.

Cell-derived matrices (CDMs) emerge as promising alternatives to conventional decellularized tissue and organ sources. They came in existence to mainly overcome the possible inflammatory/pathogenic risk associated with dECM tissues/matrices. Unlike tissue- and organ-derived ECM, CDMs are produced *in vitro* through cell-assembled ECM deposition of a fibrillar network constituted of a variety of proteins including collagen, proteoglycans, and polysaccharides. The three common methods of obtaining CDMs include monolayer cell sheet culture, template culture and pellet culture (Fig. 9). These CDMs generally require milder methods for accelerated decellularization with greater preservation of ECM components and bioactivity with effective removal of cellular debris [229,230]. Decellularized CDMs can be used as an *in vitro* model for studying cellular behavior and allow for application-specific tailoring through cell type and culture method (2D/3D, static/dynamic, culture medium, etc.). The resultant CDMs therefore contain different properties and characteristics such as protein composition, stiffness etc. depending on the culture conditions used. The utilization of cell-derived matrix technology has been summarized below in Table 5.

**Table 5:** Cell-derived ECM biomaterials/grafts developed using different cell sources.

Cells	Decellularization Method	Application	In vitro Recellularization	In vitro/*in vivo* assessment	Reference
Bone marrow mesenchymal stem cells (BMSCs) MC3T3 osteoblasts L929 fibroblasts	Five freeze-thaw cycles (liquid nitrogen and 37 °C water bath) Rinsed with sterile PBS, double distilled water and hypotonic solution DNase I treatment	Bone Tissue	Bone marrow mesenchymal stem cells (BMSCs)	Supported BMSCs proliferation and osteogenic differentiation to different extents Ectopic osteogenesis after subcutaneous implantation	[232]
Bone marrow (BM)- and adipose (AD)-derived stromal cells	PBS containing 0.5% Triton X-100 and 20 mM NH_4_OH at 37 °C Washed with PBS and sterile distilled water	Tissue specific culture system development for replicating *in vivo* niche	BM- and AD-mesenchymal stem cell (MSC)	Promoted proliferation and differentiation of MSCs, enhanced when cell origin matched CDM ECM	[233]
Coculture of Mesenchymal stem/stromal cells (MSCs) and Human umbilical vein endothelial cells (HUVECs)	0.5% Triton X‐100 containing 20 mM NH_4_OH in PBS for 5 min Washed using PBS 5 times and air-dried	Bone Tissue	Bone marrow mesenchymal stem cells	Enhanced osteogenic differentiation and angiogenic response	[234]
Adipose tissue-derived stem cells (ASCs) cultured in growth and adipogenic medium	Triton X-100 containing 20 mM (NH_4_OH) in 0.1 M glycine in PBS Washed with PBS and distilled water DNase and RNAse treatment Freeze-thaw cycles Fixed in 0.1% glutaraldehyde and treated with 0.1 M glycine in PBS	Stepwise regulation of stem cell function in adipose tissue engineering	Adipose tissue-derived stem cells	Greater migration ability cultured on growth CDM and adipogenic differentiation on adipogenic CDM	[235]
C2C12 Myoblasts at various stages of differentiation	PBS containing 0.5% Triton X-100 and 20 mM NH_4_OH PBS containing 10 mM MgCl_2_, DNase I and RNase A Treatment with 0.1% glutaraldehyde in PBS for 6 h	Muscle Tissue	C2C12 Myoblasts	Promoted myogenic differentiation in myogenic culture with early and late myogenic markers with varying levels of myogenic culture	[236]
Human lung fibroblasts	Treatment with 0.2% Triton X-100 and 10 mM NH_4_OH DNase I and RNase A treatment Washed with PBS	Expansion culture of MSCs with maintained “stemness”	Umbilical cord blood-derived MSCs (UCB-MSCs)	Enhanced cell proliferation with elongated morphology. Improved cell motility with up-regulated CXCR4 cell migration marker. Retained differentiation capacity into osteogenic lineage	[238]
Human articular chondrocytes (AC) Bone marrow stromal cells (BM)	PBS containing 0.5% Triton X-100 with 20 mM NH_4_OH Washed with PBS and deionized water	*In vitro* chondrocyte expansion with decreased dedifferentiation	Human articular chondrocytes	Faster proliferation and highest ratio of COL2A1/COL1A1 cultured on AC-CDMs compared to BM-CDMs	[242]
Synovium-derived stem cells (SDSCs) Adipose-derived stem cells Dermal fibroblasts	0.5% Triton X-100 containing 20 mM NH_4_OH	Cartilage tissue	Synovium-derived stem cells	Increased cell proliferation and increased chondrogenic markers when cultured on SDSC-CDM with lower hypertrophy potential	[243]
Human adipose-derived stem cells (hASCs)	Six freeze-thaw cycles (−80 °C and RT)	Wound dressings	L929 Fibroblasts	Supported survival and proliferation of cells *in vitro*. Improved wound healing of full-thickness skin excision in mouse model	[246]
Embryonic stem cells (ESCs)	1% Triton X-100 for 30 min DNAse I treatment Rinsed with PBS	Neural Tissue	ESC-derived neural progenitor cells	Enhanced proliferation and neural differentiation	[247]

The use of CDMs emphasizes the ability of studying cellular behavior namely migration, proliferation and differentiation as well as cellular response to ECM [231]. In one study, cell sheets were prepared by seeding BMSCs, MC3T3 pre-osteoblasts and fibroblasts on fiber mats followed by decellularization [232]. CDMs re-seeded with BMSCs promoted superior proliferation compared to TCP. Differentiation studies of the CDMs revealed that BMSC-ECM promoted stronger osteogenic differentiation as well as expressed higher presence of osteogenesis related factors VEGF and BMP-2. In another study, ECMs secreted by both BMSCs and ADSCs were used to study the effect of each culture substrate on BMSC and ADSC behavior [233]. ECM secreted from the two cell types promoted enhanced proliferation and differentiation of MSCs when cultured on its respective ECM (BMSCs on BMSC-ECM and ADSCs on ADSC-ECM), indicating the tissue-specific ECM influence on cell behavior. Furthermore, analysis of the ECM demonstrated that although the structural proteins were at similar levels, there existed unique differences in the architecture of the ECM (fiber orientation and compactness). More recently, Carvalho et al. investigated CDMs prepared from the co-culture of MSCs and HUVECs [234]. These co-culture derived matrices (Co-CDMs) supported enhanced osteogenic differentiation of hBMSCs and production of a more mature mineralized matrix as well as better angiogenic response compared to culture on CDMs derived from MSCs only, HUVECs only or TCP, suggesting the synergistic effects of Co-CDMs for facilitating ECM-like environment. These studies indicate the ability of cell-derived ECMs to guide tissue-/lineage-specific regeneration based on the matrix composition and bio-chemical cue presence, these together regulate cell behavior.

CDMs have also shown to have a conditioning effect on cells, demonstrating contact communication between CDMs and stem cells. In a recent study, Zhang et al. investigated the effects of stepwise ECM secreted components from ADSCs cultured in two different medium, namely growth and adipogenic [235]. After reseeding ADSCs on the decellularized constructs, the results of the study revealed that CDMs from ADSCs cultured in growth medium exhibited greater migration ability whereas CDMs from ADSCs cultured in adipogenic medium underwent adipogenic differentiation. Similar studies were also conducted to investigate the effects of myogenic stages of C2C12 myoblasts secreted ECM on myogenesis of stem cells [236]. Other studies investigated the influence of various stages of differentiation in regulating cellular differentiation, revealing that different stages resulted in ECM compositional differences and led to changes in cellular fate [237]. Together, these results suggest the regulatory effects of the varied secreted dECM components and can shed light on mechanisms involved in CDM mediated cellular behavior.

Numerous studies additionally support the suitability of CDMs as novel cell culture substrates for maintenance of MSC stemness and phenotypic retention/reduced dedifferentiation of chondrocytes during *ex vivo* expansion purposes with large clinical relevance in comparison to traditional culture methods [[238], [239], [240], [241]]. Chondrocyte- and BMSC-derived ECM were prepared by Zhang et al. to compare the effects of each ECM on *in vitro* expansion of human articular chondrocytes (HAC) [242]. When cultured on chondrocyte CDMs, HACs showed faster proliferation and higher maintenance of COL2A1/COL1A1 ratio. Pellet culture studies also showed cartilage-like ECM production of HAC expanded on both ECMs with better-maintained chondrocyte phenotype on chondrocyte-CDMs. In another study, decellularized CDMs derived from synovium-derived stem cells (SDSCs) demonstrated increased cellular proliferation and chondrogenic marker expression [243]. Additionally, these CDMs also showed decreased potential for hypertrophy compared to CDMs derived from ADSCs and dermal fibroblasts. As an alternative expansion substrate, human fibroblast-derived ECM (hFDM) was used for expansion of hMSCs [238]. It was seen that when cultured on these substrates, cellular proliferation and migration was significantly improved with a notable up-regulation of cell migration marker CXCR4. Differentiation studies also showed the retained differentiation capacity via gene expression and alkaline phosphatase activity.

Additionally, immortalized stem cells were used to prepare dECM and showed the ability to modulate stem cell lineage proliferation and differentiation capabilities [244]. Decellularized extracellular matrix (dECM) deposited by simian virus 40 large T antigen (SV40LT) transduced autologous infrapatellar fat pad stem cells (IPFSCs) demonstrated the ability to rejuvenate high-passage IPFSCs in proliferation and chondrogenic differentiation. Cells cultured on dECM deposited by passage 5 IPFSCs showed increased proliferation and chondrogenic differentiation capacity, however, this was reduced if cultured on dECM deposited by passage 15 IPFSCs, suggesting that passage number plays a critical role in cellular behavior when re-seeded on CDMs. CDMs were also evaluated in a rabbit osteochondral defect model. Rabbit IPFSCs were expanded on dECM deposited by human urine-derived stem cells (UDSCs) to prepare 10-day premature tissue constructs and implanted for 26 weeks [245]. The study demonstrated that UECM-expanded cells exhibited robust resurfacing effect through histological and mechanical assessment. Additionally, RNA-Seq analysis indicated that inflammation-mediated macrophage activation and polarization are potentially involved in the CDM-mediated promotion of IPFSC's chondrogenic capacity.

Although promising, CDMs with architectural, compositional, and mechanical properties similar to native counterparts are often difficult to achieve. Therefore, in order to produce mechanically relevant CDMs, composite scaffolds with biodegradable polymers have been fabricated [91]. One strategy used PLGA electrospun nanofibrous template and hADSCs to fabricate a nanofibrous dressing for wound healing [246]. Decellularized CDMs preserved type I collagen and laminin, were hydrophilic and had appropriate mechanical strength suitable for wound healing. In addition, when seeded with fibroblasts, these CDMs supported cell survival and proliferation. Crosslinking of CDMs has also been investigated for improvements in stability and resistance to rapid degradation [247]. Alternatively, biphasic calcium phosphate scaffolds with rat BMSC derived ECM were developed for a mechanically supportive and biofunctionalized scaffold [248]. The scaffolds showed increased osteoblastic differentiation of pre-osteoblasts with up-regulated osteoblastic genes: osteopontin, alkaline phosphates and BMP-2. 3D printing has also been integrated with CDMs. 3D-printed porous PCL scaffolds were populated with bone cells and cultured to allow for deposition for development of CDMs and subsequently decellularized [249]. The addition of the CDMs as part of the scaffold led to increased cellular attachment, proliferation and osteogenic activity of mesenchymal progenitor cells compared to PCL-only scaffolds. The use of CDMs is also promising for autologously derived ECMs for various applications within tissue engineering such as those mentioned previously as well as for providing a reservoir of signaling molecules/GFs [250]. In a way, a tissue biopsy or bone-marrow derived from a patient can be used as a cell source to develop a CDM that can become a graft to be implanted in the same patient. This process has merits as the patient-derived graft is being used for implantation. Patient-derived CDMs may improve favorable modulation of cellular behavior as the matrix is laid out by the same cells and limit any adverse effects that are associated with tissue-/organ-derived ECM.

Still there are differences between CDMs and tissue/organ-dECM based grafts for tissue regeneration strategies [251]. The differences lie in the key factors found within the dECM, involving the structural architecture and mechanical properties as well as matrix components such as binding motifs for cellular adhesion and signaling molecules to direct cellular behavior (Table 6). CDMs can be generated using different cellular sources, including fibroblasts, MSCs and pluripotent cells, to form a microenvironment that mimics native tissue microenvironment, however, the generation of CDMs to mimic complex tissues that require multiple cell types and their proper orientation/organization and function may be more challenging, for which tissue derived ECM may be more suited for these applications. Furthermore, the decellularization methods of CDMs and tissue-derived ECMs differ in that CDMs are prepared through *in vitro* culturing of cells for a period of time and require milder decellularization methods, whereas tissue derived ECM require more involved decellularization process, often requiring a combination of different methods. The decellularization process removes many of the foreign cellular material and antigens that may elicit immune response in order to minimize the immunogenicity risk of tissue derived ECM. CDMs, on the other hand, have the potential of being generated from autologous cells in order to produce autologous ECM scaffolds and graft systems to avoid the undesired host response altogether. However, the requirement of isolation/expansion of patient-specific cells and generation of the autologous ECM grafts is a time-consuming process compared to easily available xenogeneic donor tissues.

**Table 6:** Systematic comparison of decellularized ECM tissue with engineered matrices and cell-derived ECM.

Properties	Engineered Matrices	Cell-derived ECM	Tissue-derived ECM
Structural	☆☆	☆☆☆	☆☆☆☆☆
Biochemical/Compositional	☆☆	☆☆☆	☆☆☆☆
Binding Motifs	☆	☆☆☆	☆☆☆☆☆
Signaling Molecules	☆	☆	☆☆☆

## 11 Conclusions

Decellularized extracellular matrix (dECM) biomaterials have gained significant interest and research attention in tissue engineering and regenerative medicine over the past decade. The therapeutic potential of these biomaterials – cell, tissue and organ derived - has been realized, as evidenced by the extensive publication of over 5000 articles. FDA-approved grafts, including de-mineralized bone, skin, and ligament, further demonstrate their clinical utility as tissue substitutes. Ongoing research has led to improved methods and protocols for developing cell- and tissue-derived nonimmune biomaterials with reproducible structure and biological function. The versatility of dECM allows for the development of various biomaterial forms, such as powders, gels, sheets, 3D structures through additive manufacturing, and even tissues and organ structures with intact blood vessel structure and nerve innervation. These non-immune and bioactive biomaterials and structures outperform engineered matrices made from natural or synthetic biomaterials due to their native structural and compositional properties, including binding motifs, and biochemical signaling cues that play crucial role in host-cell interaction and guided tissue regeneration. Despite significant progress, several challenges remain. These include the need for improved cellularization methods for effective product design, a deeper understanding of how dECM influences cell behavior, and the achievement of mechanical properties that resemble those of native tissues. Standardized sterilization and preservation methods and characterization techniques need to be developed to advance decellularized tissue biomaterials and grafts for clinical translation. Additionally, the development of chemotactic dECM biomaterials that can actively promote tissue repair and regeneration without the need of respective cells and growth factors.

## Ethics approval and consent to participate

Not applicable.

## Conflict of interest statement

The authors certify that they have no affiliations with or involvement in any organization or entity with any financial interest in the subject matter or materials discussed in this manuscript.

## Conflict of interest statement

The authors declare that they have no known competing financial interests or personal relationships that could have appeared to influence the work reported in this paper.
